# High‐Durability Metal‐Doped Cu/ZnO/Al_2_O_3_ Catalysts for Reforming of Model Biomethanol

**DOI:** 10.1002/open.202600010

**Published:** 2026-05-19

**Authors:** Katsutoshi Nomoto, Hiromu Akiyama, Yasushi Sekine, Hiroki Miura, Tetsuya Shishido

**Affiliations:** ^1^ Department of Applied Chemistry for Environment Graduate School of Urban Environmental Sciences Tokyo Metropolitan University Tokyo Japan; ^2^ Department of Applied Chemistry Waseda University Tokyo Japan; ^3^ Research Center for Hydrogen Energy‐based Society Tokyo Metropolitan University Tokyo Japan

**Keywords:** autothermal reforming, bimetallic catalyst, impurities, lower alcohol, methanol

## Abstract

A series of metal‐doped Cu/ZnO/Al_2_O_3_ catalysts (M‐CZA; M = Co, Ni, Ru, Rh, Pd, Pt) were synthesized and evaluated for the autothermal reforming (ATR) of model biomass‐derived methanol (biomethanol) containing trace ethanol or 1‐butanol impurities. The objective was to improve the efficient utilization of impure biomethanol for hydrogen production. The introduction of 1 mol% ethanol decreased the methanol conversion and hydrogen production rates over CZA, Co‐CZA, Pd‐CZA, and Pt‐CZA catalysts during the initial reaction stage, with further decline over time. In contrast, Ni‐CZA, Ru‐CZA, and Rh‐CZA maintained stable activity under identical conditions. Ethanol was converted mainly into C1–C3 byproducts such as methane, acetaldehyde, and methyl acetate on CZA, Co‐CZA, Pd‐CZA, and Pt‐CZA, whereas Ni‐CZA, Ru‐CZA, and Rh‐CZA predominantly formed methane and carbon monoxide with negligible formation of carbonaceous species. Temperature‐programmed oxidation indicated the deposition of carbonaceous species on spent CZA, Co‐CZA, Pd‐CZA, and Pt‐CZA, but not on Ni‐, Ru‐, or Rh‐modified catalysts. These results suggest that Ni, Ru, and Rh enhance C–C bond cleavage in lower alcohols, thereby suppressing carbon deposition and improving catalyst durability. This study provides practical insights for designing efficient ATR catalysts for on‐site hydrogen generation from biomethanol containing impurities.

## Graphical abstract

A series of metal‐doped Cu/ZnO/Al_2_O_3_ catalysts was synthesized and evaluated for the autothermal reforming (ATR) of model biomethanol containing trace ethanol or 1‐butanol as impurities to achieve higher biomethanol utilization. The doping of Ni, Ru, and Rh into CZA suppressed carbonaceous species deposition because of their increased ability to cleave the C–C bond in impurities, resulting in improved durability in the ATR of model biomethanol.

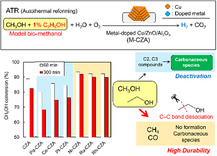

## 1 Introduction

Methanol plays a key role in carbon recycling [1, 2, 3, 4]. Methanol has been used industrially as a raw material for various chemical products and fuels and has attracted attention as a useful hydrogen carrier that can supply hydrogen to fuel cells on site because of its several advantages as a hydrogen carrier. These advantages include a lower reforming temperature (200–300°C) than that of hydrocarbons such as methane and propane, a high hydrogen/carbon (H/C) ratio, low soot formation, a relatively low boiling point, and easy and safe storage as aqueous solutions at room temperature and atmospheric pressure [3, 5, 6, 7, 8, 9]. Therefore, the demand for methanol has continuously increased [4, 10].

Methanol can be produced by the hydrogenation of carbon dioxide (CO_2_) [3, 5, 11, 12] and biomass conversion [10, 13, 14]. The transformation of methanol feedstock from conventional fossil fuels, such as natural gas and coal, to CO_2_ and biomass resources would significantly reduce CO_2_ emissions. Unlike biomass‐derived ethanol (bioethanol) produced by fermentation, biomass‐derived methanol (biomethanol) can be produced from a variety of biomass feedstocks, including inedible lignocellulosic biomass [10, 13, 15]. Biomethanol generally contains trace impurities including lower alcohols, water, aromatics, and hydrocarbons [13, 14, 16, 17, 18]. Water is produced during CO_2_ hydrogenation. In particular, the separation of lower alcohols (0%–2%) and/or water from methanol is challenging and requires energy‐intensive purification. For the reforming, since water is a source of hydrogen, the separation is unnecessary. Realizing the direct utilization of impurity‐containing biomethanol will contribute significantly to expanding the use of various biomass resources, reducing costs, and saving energy.

The direct utilization of biomethanol for reforming will accelerate the realization of carbon recycling and a hydrogen‐based society. The autothermal reforming (ATR; oxidative reforming) of methanol (Equation (1)) is an important reaction for supplying hydrogen to on‐site fuel cells [8, 19, 20, 21, 22]. Steam reforming (SR; Equation (2)) [6, 7, 8, 23, 24, 25, 26, 27] and partial oxidation (PO; Equation (3)) [3, 28, 29, 30] can be combined by simultaneously co‐feeding oxygen, methanol, and steam via ATR. In ATR, SR is promoted by the heat generated in the PO stage, reducing the external energy input. Also, fast start‐up, which is desired for mobile applications, is achieved. Cu/ZnO/Al_2_O_3_ (CZA) exhibits high activity and selectivity for SR, ATR, and water gas‐shift reactions (Equation (4)) at 200–300°C [8, 20, 31, 32, 33].

(1) $${\mathrm{CH}}_{3}\text{OH}+\left(1-n\right)H_{2}O+0.5{\mathrm{nO}}_{2}=\left(3-n\right)H_{2}+{\mathrm{CO}}_{2}\;\;\;\;\Delta {H^{0}}_{298}=+49.4-241.6n\;{\text{kJ mol}}^{‐1}$$

(2) $${\mathrm{CH}}_{3}\mathrm{OH}+H_{2}O\to 3H_{2}+{\mathrm{CO}}_{2}\;\;\;\;\Delta {H^{0}}_{298}=+49.4{\text{ kJ mol}}^{-1}$$

(3) $${\mathrm{CH}}_{3}\mathrm{OH}+0.5O_{2}\to 2H_{2}+{\mathrm{CO}}_{2}\;\;\;\;\Delta {H^{0}}_{298}=-192.2{\text{ kJ mol}}^{-1}$$

(4) $$\mathrm{CO}+H_{2}O={\mathrm{CO}}_{2}+H_{2}\;\;\;\;\Delta {H^{0}}_{298}=-41.0{\text{ kJ mol}}^{-1}$$

We recently reported that the addition of a trace amount (0.1–1.0 mol%) of lower alcohols (ethanol, 1‐propanol, or 1‐butanol) as model impurities decreased the catalytic performance (activity and durability) of CZA in SR and ATR [34]. The activity (methanol conversion and hydrogen production) in the SR of the model biomethanol (methanol with 1 mol% ethanol) using CZA, as compared with that in the SR of methanol, was significantly reduced. C2 and C3 compounds such as acetaldehyde and methyl acetate were produced as byproducts via ethanol dehydrogenation and the condensation of acetaldehyde and methanol‐derived C1 compounds, respectively. These results suggest that ethanol adsorbs on the active sites and inhibits the adsorption of methanol, resulting in a decrease in methanol conversion. In ATR, although the decrease in the initial activity was smaller than that in SR, the activity progressively decreased with the reaction time because of the deposition of carbonaceous species on CZA. The deposition of carbonaceous species can be derived from byproducts produced only in ATR, such as ethylene, acetone, and propionic acid. These results suggest that the inability of Cu‐based catalysts to cleave the C–C bond in lower alcohols is responsible for the inhibitory effects of lower alcohols.

Therefore, the dissociation of the C–C bond in lower alcohols presumably suppresses the formation of carbonaceous species, resulting in a reduction in the inhibitory effect of lower alcohols. Copper‐based catalysts convert ethanol into acetaldehyde via dehydrogenation (Equation (5)) [35, 36, 37] at temperatures below 300°C, and slightly decompose to methane and carbon monoxide (CO), including C–C bond dissociation (Equation (6)) [38, 39] above 300°C. In contrast, Cu‐based catalysts generally deactivate at temperatures above 300°C due to Cu nanoparticle aggregation. Ni‐, Co‐, and noble‐metal‐based catalysts effectively cleave the C–C bonds in lower alcohols during decomposition (Equation (6), 200–600°C) and the SR of ethanol (Equation (7), 300–800°C) [39, 40, 41, 42, 43, 44, 45, 46, 47, 48]. These reactions initially produce acetaldehyde as an intermediate via ethanol dehydrogenation (Equation (5)). The cleavage of the strong C–C bond in acetaldehyde at low temperatures (200–300°C) is rarely reported in the literature [39, 48]. Therefore, it is unclear whether Ni‐, Co‐, or noble metal‐based catalysts are effective for C–C bond dissociation at low temperatures. Furthermore, Ni‐, Co‐, and noble metal‐based catalysts are more effective for methanol decomposition (MD; Equation (8)) than for SR at 200–300°C [49, 50, 51, 52, 53]. The high selectivity of MD contributes to the high production of toxic CO and a low H/C ratio.

(5) $$C_{2}H_{5}\mathrm{OH}\to {\mathrm{CH}}_{3}\mathrm{CHO}+H_{2}\;\;\;\;\;\Delta {H^{0}}_{298}=+68.9\;{\mathrm{kJ mol}}^{-1}$$

(6) $$C_{2}H_{5}\mathrm{OH}\to {\mathrm{CH}}_{4}+\mathrm{CO}+H_{2}\;\;\;\;\Delta {H^{0}}_{298}=+49.6\;{\mathrm{kJ mol}}^{-1}$$

(7) $$C_{2}H_{5}\mathrm{OH}+3H_{2}O\to 6H_{2}+2{\mathrm{CO}}_{2}\;\;\;\;\Delta {H^{0}}_{298}=+173.5\;{\mathrm{kJ mol}}^{-1}$$

(8) $${\mathrm{CH}}_{3}\mathrm{OH}\to 2H_{2}+\mathrm{CO}\;\;\;\;\Delta {H^{0}}_{298}=+22.4\;{\mathrm{kJ mol}}^{-1}$$

Metal‐doped CZA (M‐CZA) was prepared by adding metals (M: Co, Ni, Ru, Rh, Pd, and Pt) effective for ethanol reforming to CZA. The activity and durability of M‐CZA can be improved by simultaneously promoting C–C bond dissociation and SR by doping with metals and Cu, respectively. The activity and durability of M‐CZA in the ATR of the model biomethanol were investigated. The correlation between the catalytic performance (activity, durability, and distribution of byproducts) and the structure and electronic state of each metal component (Cu and doped metal) in CZA and M‐CZA were evaluated.

## 2 Results and Discussion

### 2.1 ATR of Model Biomethanol

**FIGURE 1:**
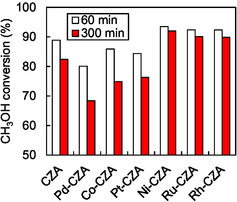
Methanol conversion in ATR of model bio‐methanol (methanol with 1mol% ethanol) over CZA and metal‐doped CZA catalysts. Reaction conditions: Catalyst 100 mg, *T*_F_ 200°C; CH_3_OH/C_2_H_5_OH/H_2_O/O_2_/N_2_ = 1.23/0.01/1.48/0.41/1.23 mmol min^−1^.

The effect of the metal doping of CZA on the catalytic performance in the ATR of model biomethanol (methanol with 1 mol% ethanol) was investigated (Figure S1). A thermocouple was installed at the top of the reactor to measure the temperature (*T*_R_) of the top of the catalyst bed. The furnace temperature (*T*_F_) was also measured, and Δ*T* was defined as the difference between *T*_R_ and *T*_F_. Figure 1 and Table 1 list the methanol conversion and H_2_ production rates of the catalysts at 200°C. Figure S2 shows the time course of methanol conversion and hydrogen production rate. The methanol conversions of Co‐CZA, Pd‐CZA, and Pt‐CZA during the initial reaction stage (60 min) were slightly lower than that of CZA. The larger crystallite size of Cu in Co‐CZA, Pd‐CZA, and Pt‐CZA than that in CZA (Table S1) may be the reason for the lower activities of Co‐CZA, Pd‐CZA, and Pt‐CZA. The methanol conversions of Co‐CZA, Pd‐CZA, and Pt‐CZA decreased progressively with increasing reaction time. The decrease in the hydrogen production rate and the increase in Δ*T* with the reaction time (Table 1) indicate that SR, an endothermic reaction, was inhibited. In contrast, the methanol conversions at the initial reaction stage with Ni‐CZA, Ru‐CZA, and Rh‐CZA were higher than that of CZA. Moreover, Ni‐CZA, Ru‐CZA, and Rh‐CZA exhibited stable methanol conversions, hydrogen production rates, and Δ*T* values for 5 h, even in methanol containing 1 mol% ethanol. The M‐CZA catalysts exhibited higher CO selectivity than CZA, regardless of the doped metal, suggesting that CO was formed via MD (Equation (8)) and/or the reverse water–gas shift reaction (Equation (4)) over the doped metal species. Consequently, the hydrogen production rates over Ni‐CZA, Ru‐CZA, and Rh‐CZA were similar to or lower than those of CZA, despite the higher methanol conversion.

**TABLE 1:** Catalytic performance in ATR of model bio‐methanol (methanol with 1 mol% ethanol).^a^

Catalyst	Methanol conversion (%)		Hydrogen production rate/mmol min^−1^		CO selectivity (%)		Δ*T*/°C	
	60 min	300 min	60 min	300 min	60 min	300 min	60 min	300 min
Rh‐CZA	92.3	89.8	2.48	2.38	25.4	27.7	87	86
Ru‐CZA	92.4	90.1	2.59	2.33	29.6	33.5	85	83
Ni‐CZA	93.5	92.0	2.49	2.46	25.5	28.8	72	74
Pt‐CZA	84.4	76.3	2.41	1.93	17.4	28.8	78	81
Co‐CZA	86.9	74.9	2.41	2.05	6.0	5.4	97	105
Pd‐CZA	80.1	68.4	2.22	1.80	20.9	27.5	96	100
CZA	88.9	82.4	2.58	2.22	3.8	2.4	73	78

a Reaction conditions: Catalyst 100 mg, *T*_F_ 200°C; CH_3_OH/C_2_H_5_OH/H_2_O/O_2_/N_2_ = 1.23/0.01/1.48/0.41/1.23 mmol min^−1^.

**FIGURE 2:**
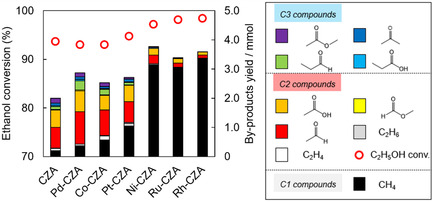
Ethanol conversion and yields of byproduct in ATR of model bio‐methanol (methanol with 1mol% ethanol). Reaction conditions: catalyst: 100 mg, 300 min, *T*_F_ 200°C; CH_3_OH/C_2_H_5_OH/H_2_O/O_2_/N_2_ = 1.23/0.01/1.48/0.41/1.23 mmol min^−1^.

Figure 2 shows the yields of the products, except for CO, in the ATR of the model biomethanol. Various C1 (methane), C2 (acetaldehyde, acetic acid, ethylene, and ethane), and C3 (methyl acetate, propionic aldehyde, acetone, and propionic acid) compounds were detected. Figure S3 shows a possible reaction path for the formation of the byproducts derived from methanol and ethanol [34]. Acetaldehyde is the primary product, and C3 compounds formed sequentially via the condensation of acetaldehyde and methanol‐derived C1 compounds [34].

C2 and C3 compounds were mainly formed over CZA, Co‐CZA, Pd‐CZA, and Pt‐CZA, whereas a small amount of methane was detected. In contrast, the yields of the C2 and C3 compounds over Ni‐CZA, Ru‐CZA, and Rh‐CZA were considerably lower than those over CZA, Co‐CZA, Pd‐CZA, and Pt‐CZA, and methane was mainly formed. Moreover, trace amounts of methane were formed over CZA, Ni‐CZA, Ru‐CZA, and Rh‐CZA during the ATR of pure methanol (without ethanol) (Figure S4), indicating that the hydrogenation of CO and CO_2_ to methane was slow. These results suggest that in the ATR of the model biomethanol, methane was formed via ethanol decomposition (Equation (6)) and acetaldehyde decomposition (Equation (9)).

(9) $${\mathrm{CH}}_{3}\mathrm{CHO}\to {\mathrm{CH}}_{4}+\mathrm{CO}\;\;\;\;\Delta {H^{0}}_{298}=-19.3{\text{ kJ mol}}^{-1}$$

During ethanol decomposition (without methanol and water), methane and acetaldehyde were formed above 200°C over Ru‐CZA and Rh‐CZA. However, methane was rarely formed over CZA, Ni‐CZA, or Pd‐CZA (Figure S5). These results indicate that ethanol decomposition occurred over Ru‐CZA and Rh‐CZA with a high capacity for C–C bond dissociation, even at low temperatures.

**FIGURE 3:**
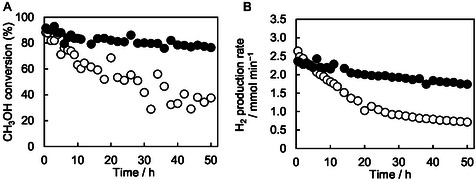
Time course of (A) methanol conversion, (B) hydrogen production rate, in the ATR of model biomethanol (methanol with 1 mol% ethanol) for 50 h over CZA (○) and Rh‐CZA (•). Reaction conditions: catalyst, 100 mg; *T*_F_, 200°C; CH_3_OH/C_2_H_5_OH/H_2_O/O_2_/N_2_ = 1.23/0.01/1.48/0.41/1.23 mmol min^−1^.

The durability tests of CZA and Rh‐CZA for a long time (50 h) were carried out in the ATR of model biomethanol at 200°C (*T*_F_) (Figure 3). The methanol conversion and H_2_ production rate of CZA monotonically decreased with reaction time and exhibited 37.6% and 0.72 mmol min^−1^ after 50 h. The lower H_2_ production‐to‐methanol conversion ratio indicates that methanol was consumed via total oxidation, and SR hardly proceeded. The initial (1 h) methanol conversion and H_2_ production rate of Rh‐CZA were 90.5% and 2.34 mmol min^−1^, and the methanol conversion and H_2_ production rate after 50 h were 76.5% and 1.74 mmol min^−1^, respectively. Consequently, Rh‐CZA exhibited remarkably improved durability compared to CZA.

### 2.2 Characterization of Catalysts

**FIGURE 4:**
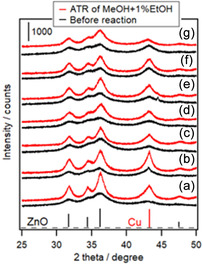
XRD patterns of Cu‐based catalysts before and after ATR of model bio‐methanol (methanol with 1 mol% ethanol). (a) CZA, (b) Pd‐CZA, (c) Co‐CZA, (d) Pt‐CZA, (e) Ni‐CZA, (f) Ru‐CZA, and (g) Rh‐CZA. Reaction conditions: 300 min, *T*_F_ 200°C; CH_3_OH/C_2_H_5_OH/H_2_O/O_2_/N_2_ = 1.23/0.01/1.48/0.41/1.23 mmol min^−1^. Before reaction: 300°C, 1 h, H_2_ 10 mL min^−1^.

Figure 4 shows the XRD patterns of the CZA and M‐CZA catalysts before and after the ATR of the model biomethanol. The diffraction peaks were attributed only to Cu and ZnO, except for those of Ni‐CZA, indicating that the doped metal was highly dispersed in CZA. The Cu and ZnO diffraction peaks became sharper after ATR, suggesting that the aggregation of Cu and ZnO caused catalyst deactivation. Notably, the aggregation of Cu and ZnO was also observed in Ni‐CZA, Ru‐CZA, and Rh‐CZA, which exhibited high durability in the ATR of biomethanol (Table S1). Thus, Cu and ZnO aggregation was not the primary reason for the gradual methanol conversion decrease with the reaction time. For M‐CZA catalysts, the change in the intensity of diffraction lines due to Cu metal before and after the ATR reaction is smaller compared with the CZA catalyst. This is thought to result from dopants altering the physical properties of the Cu metal nanoparticles, such as their melting point, thereby enhancing their stability and suppressing the aggregation of Cu‐M alloy (M: dopants) nanoparticles.

**FIGURE 5:**
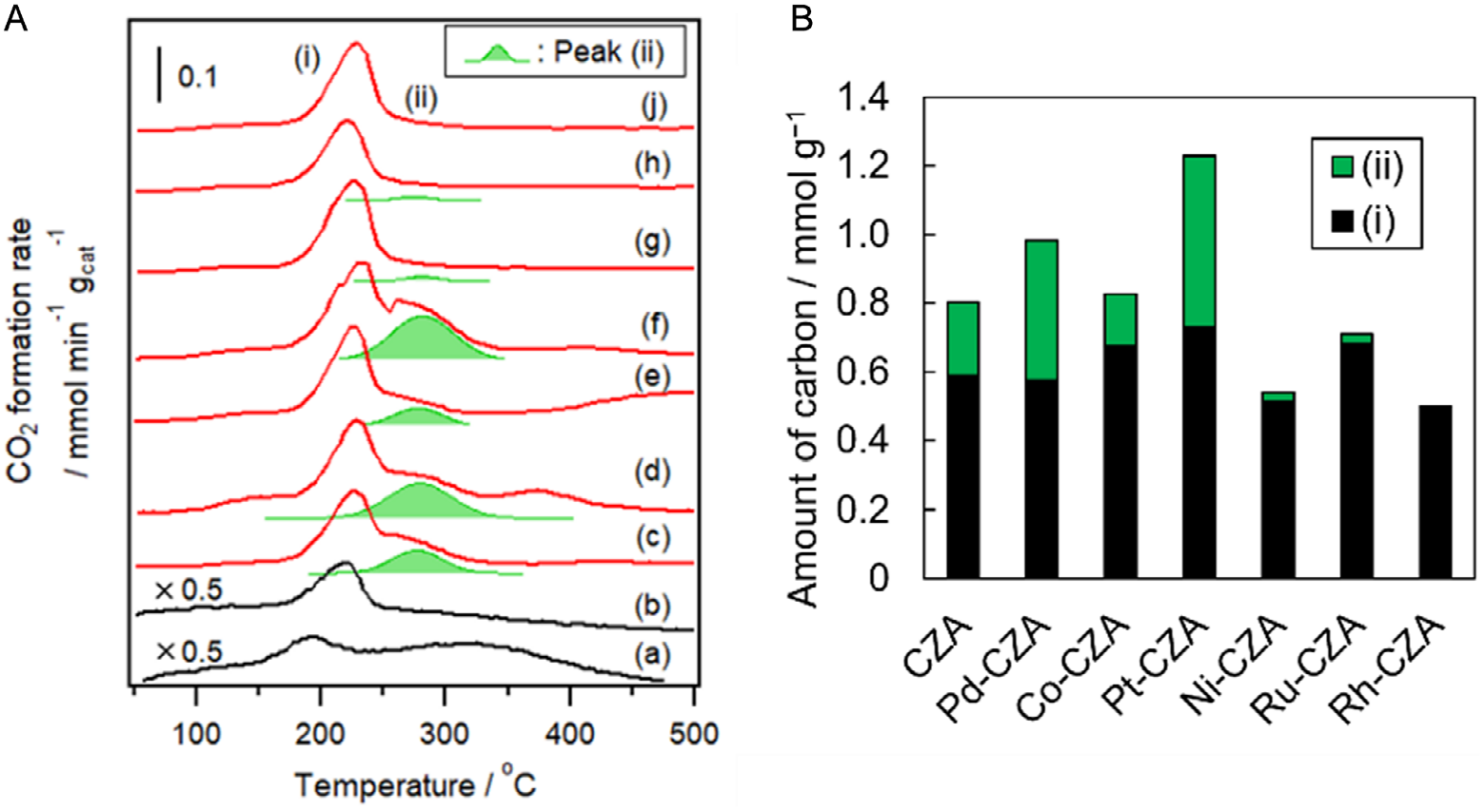
(A) TPO profiles of spent CZA and metal‐doped CZA after the ATR of model biomethanol (methanol with 1 mol% ethanol) (red) and fresh CZA treated with methanol or ethanol (black). (B) The amount of carbonaceous species formed on the spent catalysts. (a) CZA, (b) Pd‐CZA, (c) Co‐CZA, (d) Pt‐CZA, (e) Ni‐CZA, (f) Ru‐CZA, and (g) Rh‐CZA. Reaction conditions: 300 min, *T*_F_ 200°C; CH_3_OH/C_2_H_5_OH/H_2_O/O_2_/N_2_ = 1.23/0.01/1.48/0.41/1.23 mmol min^−1^. Before reaction: 300°C, 1 h, H_2_ 10 mL min^−1^. Methanol or ethanol treatment: CZA was treated with CH_3_OH/He or C_2_H_5_OH/He at 40°C for 1 h, and then CZA was purged with He at 40°C for 1 h. The values ((i) and (ii)) were estimated from the areas of peak (i) and (ii) shown in Figure 5A.

Carbon deposition often causes CZA deactivation in the ATR of the model biomethanol [45, 46, 47]. Figure 5 shows the TPO profiles of CZA and M‐CZA after the ATR of the model biomethanol (methanol with 1 mol% ethanol) for 5 h. Co‐CZA, Pd‐CZA, Pt‐CZA, and CZA exhibited the two CO_2_ production peaks ((i) and (ii)) at 225 and 280°C, respectively. The temperature of peak (i) (220°C) was close to that observed in the TPO profile of CZA treated with ethanol at 40°C, while peak (ii) was not detected on spent CZA after the ATR of methanol or ethanol (without impurities) [40, 45, 46]. Peaks (i) and (ii) are attributed to the ethanol and byproduct (C3 compounds)‐derived carbonaceous species, respectively [34]. In contrast, Ni‐CZA, Ru‐CZA, or Rh‐CZA did not exhibit a peak (ii). These results suggest that C–C bond dissociation contributed not only to the decrease in byproduct yields (C3 compounds), but also to the suppression of carbonaceous species deposition (peak (ii)), resulting in the high durability of Ni‐CZA, Ru‐CZA, and Rh‐CZA. Catalysts with a high C–C bond dissociation ability, especially Ni‐based catalysts, generally form coke in ethanol reforming at relatively low temperatures (>300°C) via the Boudard reaction and reverse carbon gasification (Equations (10) and (11), respectively) [54]. In contrast, Ni‐CZA, Ru‐CZA, and Rh‐CZA rarely formed coke in the ATR of the model biomethanol, which is detected in the TPO profiles at temperatures above 300°C. This is due to several reasons, such as (i) lower reaction temperatures than that for ethanol reforming below 300°C, (ii) ensemble effect (e.g., small particle sizes and alloy formation; aforementioned), suppressing coke formation, and (iii) low amount of ethanol, that is, a high H_2_O/ethanol and O_2_/ethanol ratio, promoting coke oxidation by H_2_O and/or O_2_.

(10) $$2\mathrm{CO}\to C+{\mathrm{CO}}_{2}\;\;\;\;\;\Delta {H^{0}}_{298}=-172.6{\text{ kJ mol}}^{-1}$$

(11) CO+H2→C+H2O ΔH0298=−131.6 kJ mol−1

To gain further insight into the local structures and electronic states of Cu and the doped metals in M‐CZA, the catalyst was characterized using XAS. The X‐ray absorption near edge structure (XANES) spectra were measured to investigate the electronic state and local structure of each metal component in M‐CZA. The shapes of the Cu K‐edge XANES spectra and the first derivative of the M‐CZA XANES spectra after hydrogen reduction were similar to those of CZA (Figure S6), whereas they were different from those of the Cu foil, Cu_2_O, and CuO. More specifically, the CZA and M‐CZA pre‐edge peaks appeared at 8981 eV, which is higher than that for Cu foil (Cu^0^, 8980 eV) and lower than those for Cu_2_O (Cu^+^, 8982 eV) and CuO (Cu^2+^, 8984 eV). These results indicate that the Cu species in both CZA and M‐CZA existed as a mixture of Cu^0^ and Cu^+^. The Cu K‐edge *k* ^3^‐weighted extended X‐ray absorption fine structure (EXAFS) oscillations of CZA and M‐CZA were similar to those of the Cu foil (Figure S7). The intensities of the oscillations were weaker than those of the Cu foil. Moreover, the peak positions in the Fourier transforms (FTs) of the EXAFS spectra of the CZA and M‐CZA catalysts were similar to that of Cu foil (2.2 Å), which is assignable to the Cu–Cu bond. The height of this peak was lower than that of the Cu foil. These results indicate that Cu metal nanoparticles were dispersed in CZA and M‐CZA. Curve‐fitting analyses of the EXAFS spectra of CZA and M‐CZA were also performed, and their structural parameters are summarized in Table S2. The coordination numbers of the Cu–Cu bond of CZA and M‐CZA were 5.1–6.9, indicating that Cu metal nanoparticles were dispersed in CZA and M‐CZA.

The XANES spectra, EXAFS oscillations, and FTs of the EXAFS spectra of each doped metal (Co, Ni, Rh, and Pd K‐edges) in M‐CZA are shown in Figures S8–S15. The edge positions of the Co, Ni, Rh, and Pd K‐edge XANES spectra and their first derivatives of M‐CZA were similar to those of the Co, Ni, Rh, and Pd foils (Figures S8, S10, S12, and S14), indicating that Co, Ni, Rh, and Pd were present in the metallic state. However, the position and intensity of the white lines of Co‐CZA, Ni‐CZA, Rh‐CZA, and Pd‐CZA were slightly different from those of the Co, Ni, Rh, and Pd foils, thereby indicating that electronic interactions between the doped metal (Co, Ni, Rh, and Pd) and Cu occurred through the alloying of the doped metal and Cu.

The electronic interactions were observed using X‐ray photoelectron spectroscopy (XPS) (Figure S16 and Table S3). The XPS spectra of the Rh 3d and Pd 3d regions of Rh‐CZA and Pd‐CZA shifted to lower binding energies than those of Rh/ZnO and Pd/ZnO, respectively, whereas the spectra of the Cu 2p regions of Rh‐CZA and Pd‐CZA shifted slightly to higher binding energies than those of CZA. These results indicate that electrons were transferred from Cu to the doped metals (Pd and Rh) in Pd‐CZA and Rh‐CZA. Additionally, The surface Pd and Rh composition ratios normalized by the Cu concentration in Pd‐CZA and Rh‐CZA were estimated from the XPS spectra to be 0.06 and 0.05, respectively (Table S4). These values are higher than the ratio of the nominal Pd and Rh loadings per Cu (Pd/Cu = Rh/Cu = 0.015), suggesting that the doped metals (Pd and Rh) were abundant on the surface.

The Rh and Pd K‐edge *k* ^3^‐weighted EXAFS oscillations (Figures S13 and S15) of Rh‐CZA and Pd‐CZA differed from those of the Rh and Pd foils, and the intensity of the oscillations was significantly weaker. The peaks located at 2.4 Å in the FTs of the Rh and Pd K‐edge *k* ^3^‐weighted EXAFS spectra of the Rh and Pd foils were attributed to the Rh–Rh and Pd–Pd linkages, respectively. Rh‐CZA and Pd‐CZA exhibited weaker and broader peaks at 2.2 Å, which is shorter than that attributed to the Rh–Rh and Pd–Pd linkages. Table 2 summarizes the structural parameters estimated by the curve‐fitting analyses of each doped metal K‐edge EXAFS spectrum. The EXAFS data for Rh‐CZA and Pd‐CZA fit well upon assuming a Rh–Cu and Pd–Cu shell, indicating the formation of Rh–Cu and Pd–Cu alloys, in which Rh and Pd atoms exist in an isolated state and are surrounded by Cu atoms. The longer bond distance of the Rh–Cu and Pd–Cu linkages than that of the Cu–Cu linkage was due to the larger size of Rh (1.42 Å) and Pd (1.39 Å) than that of Cu (1.32 Å).

**TABLE 2:** Curve‐fitting analyses of the Co, Ni, Rh, and Pd K‐edge EXAFS spectra.^a^

Catalyst^b^	Scatter	N^c^	*R* d (Å)	Δ*E* e (eV)	D.W.^f^
Rh‐CZA	Rh–Cu	5.4	2.56	−8.41	0.086
Ni‐CZA	Ni–Cu	8.7	2.47	−8.98	0.082
Co‐CZA	Co–Cu	6.3	2.48	−10.41	0.104
Pd‐CZA	Pd–Cu	6.4	2.57	−7.30	0.085

a FEFF8 was used to calculate the backscattering amplitude and phase‐shift functions [54]. The crystal structure parameters of RhCu (Fm‐3 m: 01‐074−578), CuNi (Fm‐3 m: 01‐071−783), Co0.52Cu0.48 (Fm‐3 m: 00‐050−145), and PdCu (Fm‐3 m: 00‐048−155) were obtained from the International Center for Diffraction Data.

b After H_2_ reduction (300°C, 1 hr, and H_2_ 10 mL min^−1^).

c Number of coordinates.

d Distance.

e Edge shift.

f Debye‐Waller factor.

The Co and Ni K‐edge *k* ^3^‐weighted EXAFS oscillations (Figures S9 and S11) of Co‐CZA and Ni‐CZA were similar to those of the Co and Ni foils, and the intensity of the oscillations was significantly weaker. The peaks located at 2.2 Å in the FTs of the Co and Ni K‐edge *k* ^3^‐weighted EXAFS spectra of the Co and Ni foils were attributed to the Co–Co and Ni−Ni linkages, respectively. The EXAFS data for Co‐CZA and Ni‐CZA fit well upon the assumption of Co–Cu and Ni–Cu shells (Table 2), indicating the formation of Co–Cu and Ni–Cu alloys. The Co and Ni atoms likely exist in an isolated state and are surrounded by Cu atoms. The shorter bond distance of the Co–Cu and Ni–Cu linkages was consistent with the smaller size of Co (1.24 Å) and Ni (1.25 Å) than that of Cu (1.32 Å).

These characterizations indicate that the structure of the alloy and the electronic state of Cu on M‐CZA were similar, regardless of the doped metal. Therefore, the ability of M‐CZA to inhibit the precipitation of carbonaceous species was strongly dependent on the C—C bond dissociation ability of the doped metal [39, 40, 41, 45, 46, 55].

### 2.3 Effect of the Reaction Conditions

**FIGURE 6:**
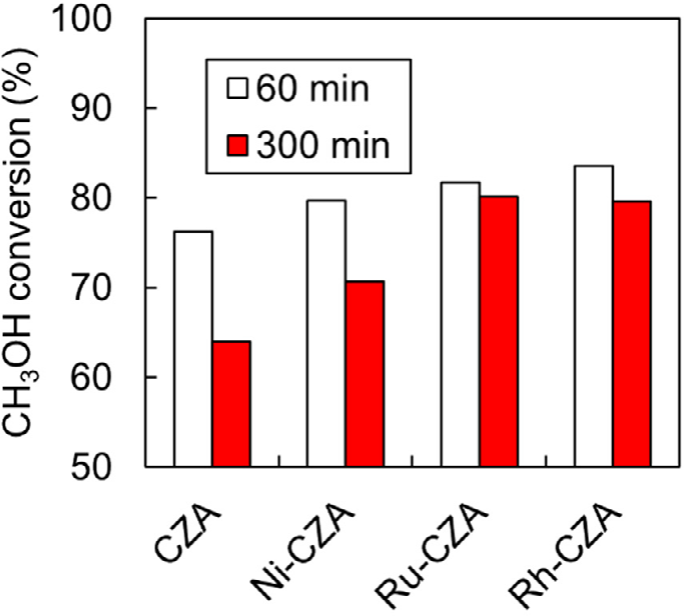
Methanol conversion in ATR of model bio‐methanol (methanol with 1mol% ethanol) over CZA and metal‐doped CZA at 140°C. Reaction conditions: Catalyst: 100 mg; CH_3_OH/C_2_H_5_OH/H_2_O/O_2_/N_2_ = 1.23/0.01/1.48/0.41/1.23 mmol min^−1^.

The effect of the *T*_F_ on the catalytic activity and durability of Ni‐CZA, Ru‐CZA, and Rh‐CZA in the ATR of the model biomethanol (methanol with 1 mol% ethanol) was investigated. The initial methanol conversions for CZA, Ni‐CZA, Ru‐CZA, and Rh‐CZA at 140°C were approximately 10% lower (Figure 6) than those at 200°C (Figure 1). The methanol conversions over Ru‐CZA and Rh‐CZA decreased slightly (within 5% of the initial conversion). In contrast, the methanol conversions over Ni‐CZA and CZA decreased (approximately 12.7% of the initial conversion). The methane yield over Ni‐CZA decreased significantly at 140°C (Figure S17), as compared with that at 200°C (Figure 2). The methane yields over Ru‐CZA and Rh‐CZA decreased slightly.

**FIGURE 7:**
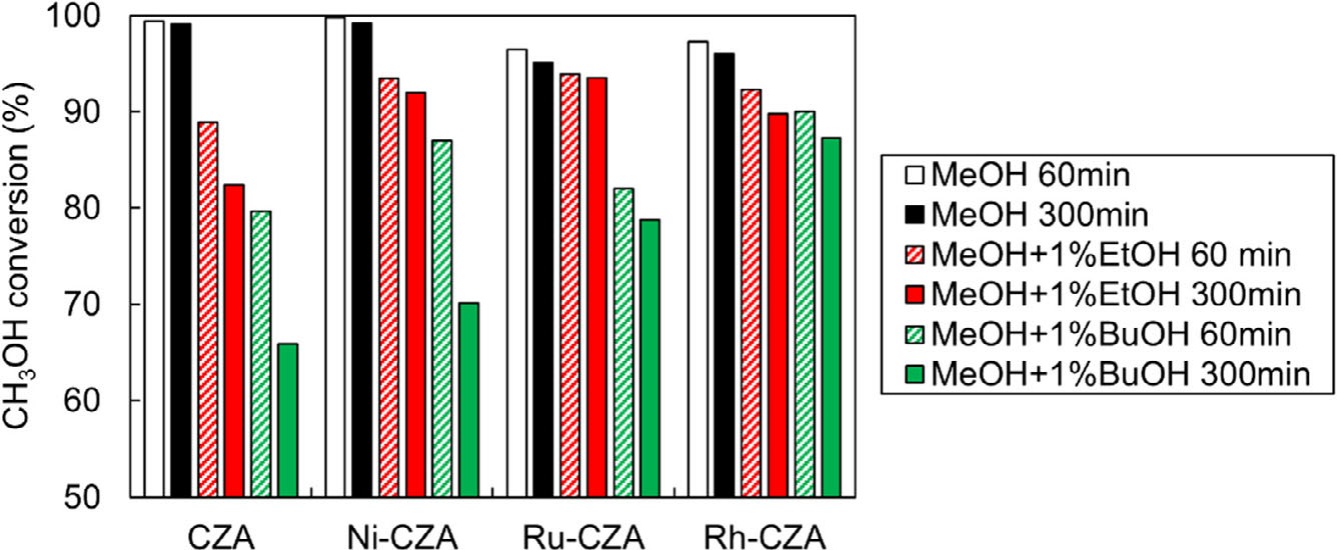
Methanol conversion in reforming of methanol with 1mol% lower alcohols. Catalyst: 100 mg, *T*_F_ 200°C, CH_3_OH(/Alcohol)/H_2_O/O_2_/N_2_ = 1.23(/0.01)/1.48/0.41/1.23 mmol min^−1^.

The methanol conversions in the ATR of the model biomethanol containing ethanol and 1‐butanol with different carbon numbers as model impurities were compared (Figure 7). Ru‐CZA and Rh‐CZA exhibited stable methanol conversion for 5 h, even in the ATR of methanol with 1 mol% 1‐butanol, although the methanol conversion was slightly lower than that of methanol with 1 mol% ethanol. The methanol conversion of Ni‐CZA decreased progressively, similar to that of CZA. The XRD patterns of CZA, Ni‐CZA, Ru‐CZA, and Rh‐CZA after the reaction (Figure S18) showed no significant structural changes, regardless of the carbon number of the lower alcohols. The TPO profiles of the catalysts after ATR indicate that peak (ii) at approximately 280°C disappeared upon the addition of Ni, Ru, and Rh to CZA even in the presence of 1‐butanol (Figure S19). However, a new peak was detected at 450°C in the TPO profile of Ni‐CZA, indicating the deposition of new carbonaceous species on Ni‐CZA. Therefore, Ru‐CZA and Rh‐CZA exhibited high durability even in the ATR of methanol with 1‐butanol because of their high ability to cleave the C–C bonds in 1‐butanol. Ni is cost‐effective compared to Ru and Rh due to its lower price. However, at low temperature (*T*_F_: 140°C) or in the presence of 1‐butanol, its activity does not improve compared with undoped catalysts. These disadvantages are considered compared with Ru and Rh.

A physical mixture composed of CZA and ZnO/Al_2_O_3_ (ZA)‐supported Ru or Rh catalysts was prepared and applied to the ATR of a model biomethanol to evaluate its ability to reduce the inhibitory effects of ethanol. ZA was prepared using the same procedure as that used to prepare CZA, except that the Cu precursor was not used.

**FIGURE 8:**
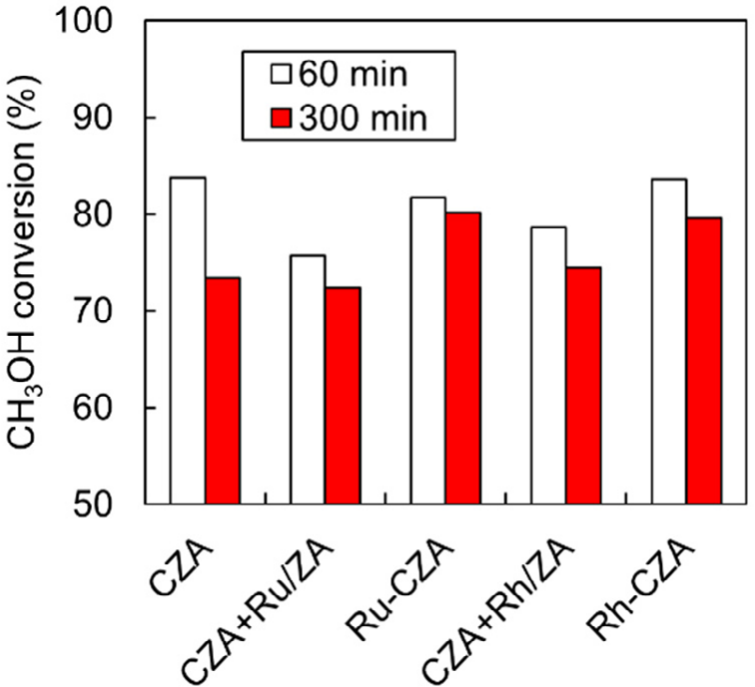
Methanol conversion in ATR of model bio‐methanol (methanol with 1mol% ethanol) over physical mixture at 140°C. Reaction conditions: Catalyst: single 100 mg, physical mixture CZA (100 mg)+Ru/ZA or Rh/ZA (100 mg); CH_3_OH/C_2_H_5_OH/H_2_O/O_2_/N_2_ = 1.23/0.01/1.48/0.41/1.23 mmol min^−1^.

The physical mixtures (CZA + Ru/ZA and CZA + Rh/ZA) exhibited lower methanol conversion at 140°C (*T*_F_) than Ru‐CZA and Rh‐CZA (Figure 8). Additionally, the physical mixture exhibited a slightly lower ethanol conversion and methane yield than Ru‐CZA and Rh‐CZA (Figure S20). These results suggest that the intimate contact of Ru and Rh with metallic Cu species (Ru–Cu and Rh–Cu alloys) can act as active sites for the decomposition of ethanol and acetaldehyde, resulting in the suppression of the deposition of carbonaceous species on the Cu surface.

## 3 Conclusion

A series of M‐CZA (M: Co, Ni, Ru, Rh, Pd, and Pt) catalysts were prepared and tested for the ATR of model biomethanol containing trace ethanol or 1‐butanol as impurities to achieve higher biomethanol utilization. The doping of Ni, Ru, and Rh into CZA suppressed carbonaceous species deposition because of their increased ability to cleave the C–C bond in ethanol and 1‐butanol, resulting in the improved durability of Ni‐CZA, Ru‐CZA, and Rh‐CZA in the ATR of model biomethanol. In contrast, the activities (methanol conversion and hydrogen production rate) of CZA, Co‐CZA, Pd‐CZA, and Pt‐CZA decreased during the initial stage of the reaction and progressively decreased with the reaction time. Co‐CZA, Pd‐CZA, and Pt‐CZA, similar to CZA, converted ethanol into various C1, C2, and C3 byproducts, such as methane, acetaldehyde, and methyl acetate, resulting in the deposition of carbonaceous species. The structural characterization and catalytic performance of the physical mixture indicated that the formation of alloys such as Cu–Rh and Cu–Pd promotes the conversion of lower alcohols on the Cu surface into gaseous products. This study provides valuable insights into catalyst design with significant implications for on‐site hydrogen production, particularly for the transformation of biomethanol containing impurities.

## 4 Experimental Section

### 4.1 Catalyst Preparation

The CZA catalyst was prepared using a coprecipitation method [56]. An aqueous solution of metal nitrate (0.3 M) was added dropwise to an aqueous solution of Na_2_CO_3_ (0.3 M) under vigorous stirring for 0.5 h, and the resulting solution was aged at 50°C for 20 h. The resulting precipitate was washed with distilled water in a centrifuge, dried at 80°C for 20 h in an oven, and calcined at 300°C for 3 h in flowing air. The Cu/Zn/Al molar ratio of the CZA catalyst was 45/45/10.

M‐CZA (M: Co, Ni, Ru, Rh, Pd, and Pt) catalysts were prepared using an impregnation method. The prepared CZA was stirred in an acetone solution of metal precursors (nickel(II) acetate tetrahydrate, cobalt(II) acetate tetrahydrate, palladium(II) acetate, platinum(II) bis(acetylacetonate), ruthenium(III) acetylacetonate, or rhodium(III) acetylacetonate) at 25°C for 2 h. The samples were evaporated at 25°C, dried at 80°C for 20 h in an oven, and calcined at 300°C for 3 h in air. The loading of the doped metal in M‐CZA was 1 wt%.

### 4.2 Catalytic Test

Methanol and model biomethanol were autothermally and steam‐reformed using a fixed‐bed flow reactor at atmospheric pressure (Figure S1). A thermocouple was installed at the top of the reactor to measure the temperature (*T*_R_) of the top of the catalyst bed. The furnace temperature (*T*_F_) was also measured, and Δ*T* was defined as the difference between *T*_R_ and *T*_F_. The catalysts were reduced at 300°C (*T*_F_) for 1 h under 14.3 vol% H_2_ diluted with N_2_ (35 mL min^−1^) and then cooled to reaction temperature for 1 h under N_2_ (30 mL min^−1^). An aqueous solution of methanol or model biomethanol (methanol with lower alcohols) was fed using a HPLC pump (PU‐980, JASCO, Japan) and evaporated prior to the catalyst bed at 120°C. The feed gas compositions were CH_3_OH/H_2_O/N_2_ = 1.23/1.48/1.23 mmol min^−1^ (30/36/30 mL min^−1^) for SR and CH_3_OH/H_2_O/O_2_/N_2_ = 1.23/1.48/0.41/1.23 mmol min^−1^ (30/36/10/30 mL min^−1^) for ATR. The products were analyzed using two online and one offline gas chromatographs (GCs). An online GC (GC‐8A, SHIMADZU, Japan) equipped with a packed Molecular Sieve 5A column and a thermal conductivity detector was used to analyze H_2_, O_2_, N_2_ and CO with Ar as the carrier gas. The other online GC equipped with a packed Porapak‐Q column, flame ionization detector (FID), and methanizer (MTN‐1, SHIMADZU, Japan) was used to analyze CH_3_OH, CO_2_, CO, CH_4_, C_2_H_4_, and C_2_H_6_ with N_2_ as the carrier gas. The liquid products collected in a cold trap (0°C) were analyzed using an offline GC (GC‐2014, SHIMADZU, Japan) equipped with a DB‐FFAP column and a FID. The methanol and ethanol conversions and their selectivity for CO were determined using Equations (12) and (13):

(12) $$\left(\text{Conversion of}\;X\right)\left(\%\right)=\frac{N_{X}^{in}-N_{X}^{out}}{N_{X}^{in}}\times 100$$

(13) $$\left(\text{CO selectivity}\right)\left(\%\right)=\frac{N_{\mathrm{CO}}^{out}}{N_{\mathrm{CO}2}^{out}+N_{\mathrm{CO}}^{out}+N_{\mathrm{CH}4}^{out}}\times 100$$

where $N_{X}^{in}$ is the initial number of moles of X (methanol and ethanol), $N_{X}^{out}$ is the outlet gas of X (methanol and ethanol), and $N_{P}^{out}$ is the number of moles of the product.

### 4.3 Characterization

The X‐ray diffraction (XRD) patterns of the catalysts were recorded using a Rigaku SmartLab diffractometer with Cu Kα radiation. The samples were scanned in the 2*θ* range of 25–50° at a rate of 3° min^−1^ and resolution of 0.002°.

Temperature‐programmed oxidation (TPO) profiles were recorded using a BELCAT II instrument (MicrotracBEL, Japan) to determine the quantity of carbonaceous material deposited on the spent catalysts during the reaction. Prior to performing the TPO measurements, the catalysts were purged at 25°C for 2 h under a He flow (30 mL min^−1^). Subsequently, the catalysts were heated in 20 vol% O_2_ diluted with He (50 mL min^−1^) from 25 to 800°C at a heating rate of 10°C min^−1^. The amount of carbonaceous material was estimated from the CO_2_ formation (m/*z* = 44) quantified using a Q‐mass mass spectrometer (BELMass, MicrotracBEL, Japan) and the absolute calibration curve method. The TPO values of the catalysts treated with methanol and ethanol were also measured. Prior to the TPO measurements, the fresh catalyst was reduced at 300°C for 1 h under 16.7 vol% H_2_ diluted with He (30 mL min^−1^) and then cooled to 40°C under He (30 mL min^−1^). The samples treated with 8 vol% methanol or 12 vol% ethanol under a He flow (30 mL min^−1^) at 40°C for 1 h were then flushed with He (30 mL min^−1^) for 1 h. Subsequently, the TPO measurements were performed using the same procedure as mentioned above.

X‐ray absorption spectroscopy (XAS) was performed using the BL01B1 beamline at the SPring‐8 (Hyogo, Japan) with a ring energy of 8 GeV and a stored current of 99.5 mA. The catalysts were reduced at 300°C for 1 h under H_2_ (10 mL min^−1^) and then stored under N_2_ in a glove box to avoid exposure to air. The Cu K‐edge (8.90 keV), Co K‐edge (7.71 keV), and Ni K‐edge (8.33 keV) X‐ray absorption spectra were recorded using a Si(111) crystal monochromator, whereas the Rh K‐edge (23.22 keV) and Pd K‐edge (24.34 keV) X‐ray absorption spectra were recorded using a Si(311) crystal monochromator. The spectra of the reference samples and the Cu and Ni K‐edges of the catalysts were obtained in the transmittance mode, while the Co, Rh, and Pd K‐edge spectra of the catalysts were obtained in the fluorescence mode. Data reduction was performed using xTunes (Science and Technology Institute, Co.) [57].

## Funding

This study was supported by the Ministry of Education, Culture, Sports, Science and Technology (22H01870) and the Tokyo Metropolitan Government (R3‐1).

## Conflicts of Interest

The authors declare no conflicts of interest.

## Supporting information

Supplementary Material

## Data Availability

The data that support the findings of this study are available from the corresponding author upon reasonable request.
