# Supplementary material for: High‐Durability Metal‐Doped Cu/ZnO/Al2O3 Catalysts for Reforming of Model Biomethanol
Source: ChemistryOpen. 2026 May 19;15(6):e202600010. doi: 10.1002/open.202600010 (PMC13184550; doi:10.1002/open.202600010)
Supplement: Supplementary file 1 — Supplementary Material [file OPEN-15-e202600010-s001.pdf]

Supporting information

High-Durability Metal-Doped $Cu/ZnO/Al_2O_3$ Catalysts for Reforming of Model Biomethanol

Katsutoshi Nomoto[a], Hiromu Akiyama[b], Yasushi Sekine[b], Hiroki Miura[a, c], Tetsuya Shishido[a, c]*

[a] Department of Applied Chemistry for Environment, Graduate School of Urban Environmental Sciences, Tokyo Metropolitan University, 1-1 Minami-Osawa, Hachioji, Tokyo 192-0397, Japan.

[b] Department of Applied Chemistry, Waseda University, 3-4-1 Okubo, Shinjuku, Tokyo 169-8555, Japan.

[c] Research Center for Hydrogen Energy-based Society, Tokyo Metropolitan University, 1-1 Minami-Osawa, Hachioji, Tokyo 192-0397, Japan.

*Corresponding author. E-mail: shishido-tetsuya@tmu.ac.jp

Experimental

1.1. Materials

Methanol (99.8%), ethanol (99.5%), n-butanol (99.0%), copper(II) nitrate trihydrate (100%), zinc(II) nitrate hexahydrate (99.0%), aluminum(III) nitrate nonahydrate (98.0%), cobalt(II) acetate tetrahydrate (99.0%), nickel(II) acetate tetrahydrate (98.0%), palladium(II) acetate (97.0%), platinum(II) bis(acetylacetonate) (48.6–50.6% as Pt), sodium carbonate (99.8%), acetone (99.0%), and boron nitride (100%) were purchased from FUJIFILM Wako Pure Chemical Corporation (Japan). Palladium(II) acetylacetonate (97%), ruthenium(III) acetylacetonate (97%) and rhodium(III) acetylacetonate (97%) were purchased from Sigma-Aldrich (USA). ZnO (FZO-50) was purchased from Ishihara Sangyo Kaisha Ltd., and calcined at 300 °C for 3 h flowing air prior to the preparation of catalysts.

1.2. Preparation of ZnO supported noble metal catalyst

ZnO supported noble metal catalysts (noble metal: Rh and Pd) were prepared via an impregnation method. The ZnO was stirred in an acetone solution of metal precursor (palladium(II) acetylacetonate or ruthenium(III) acetylacetonate) at 25 °C for 2 h. The sample was evaporated at 25 °C, dried at 80 °C for 20 h in an oven, and then calcined at 300 °C for 3 h flowing air. The loading amount of noble metal in Pd/ZnO and Rh/ZnO was 1wt%.

1.3. Characterization

The copper metal surface area was determined according to the N_2O decomposition method using a BELCAT II (MicrotracBEL, Osaka, Japan). Prior to performing this measurement, the sample was reduced at 300 °C for 1 h in a mixed gas flow of H_2 and N_2 (5/25 mL min^{-1}) and cooled to 90 °C under He flow (30 mL min^{-1}). A pulse (1 mL) of 5 vol% N_2O/He was repeatedly introduced into the sample, and the consumption of N_2O was detected by a thermal conductivity detector (TCD) and a packed SHINCARBON ST column with He as the carrier gas. As previously reported, a reaction stoichiometry consisting of two Cu atoms per O atom and a Cu surface density of 1.68×10^{19} Cu_{atoms} m^{-2} was assumed [1].

The Brunauer-Emmett-Teller (BET) specific surface area was estimated from the N_2 adsorption isotherms obtained using a BELSORP-mini II (MicrotracBEL Corp.) at −196 °C after evacuation of the sample at 300 °C for 3 h.

X-ray photoelectron spectroscopy (XPS) measurements were carried out on VersaProbe 4 (ULVAC-PHI Inc.) using Al Kα radiation (1486.6 eV, 15 kV, 100 µA) and equipped with an infrared lamp heating chamber (Advanced Riko Inc.). The binding energies were calibrated by using the Zn $2p_{3/2}$ signal at 1021.80 eV [2]. The mixture of sample and boron nitride was grinded by agate, and was formed into dicks by pressurizing at 4 MPa. The disk sample was reduced for 0.5 h in a mixed gas flow of H_2 and Ar (10/30 mL min^{-1}) at atmospheric pressure in a heating chamber. After depressurizing the heating chamber, the reduced sample was transferred to an analysis chamber without exposure to air.

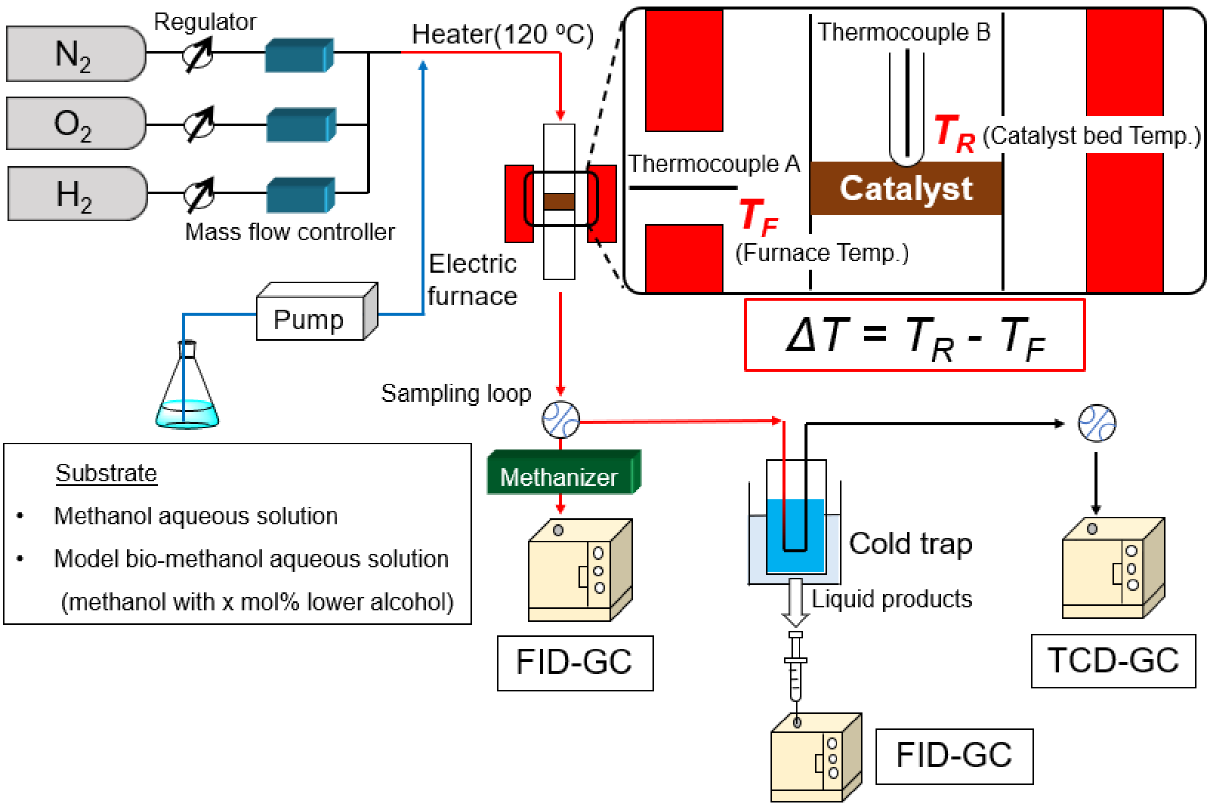

Fig. S1. Reaction system for reforming of methanol.

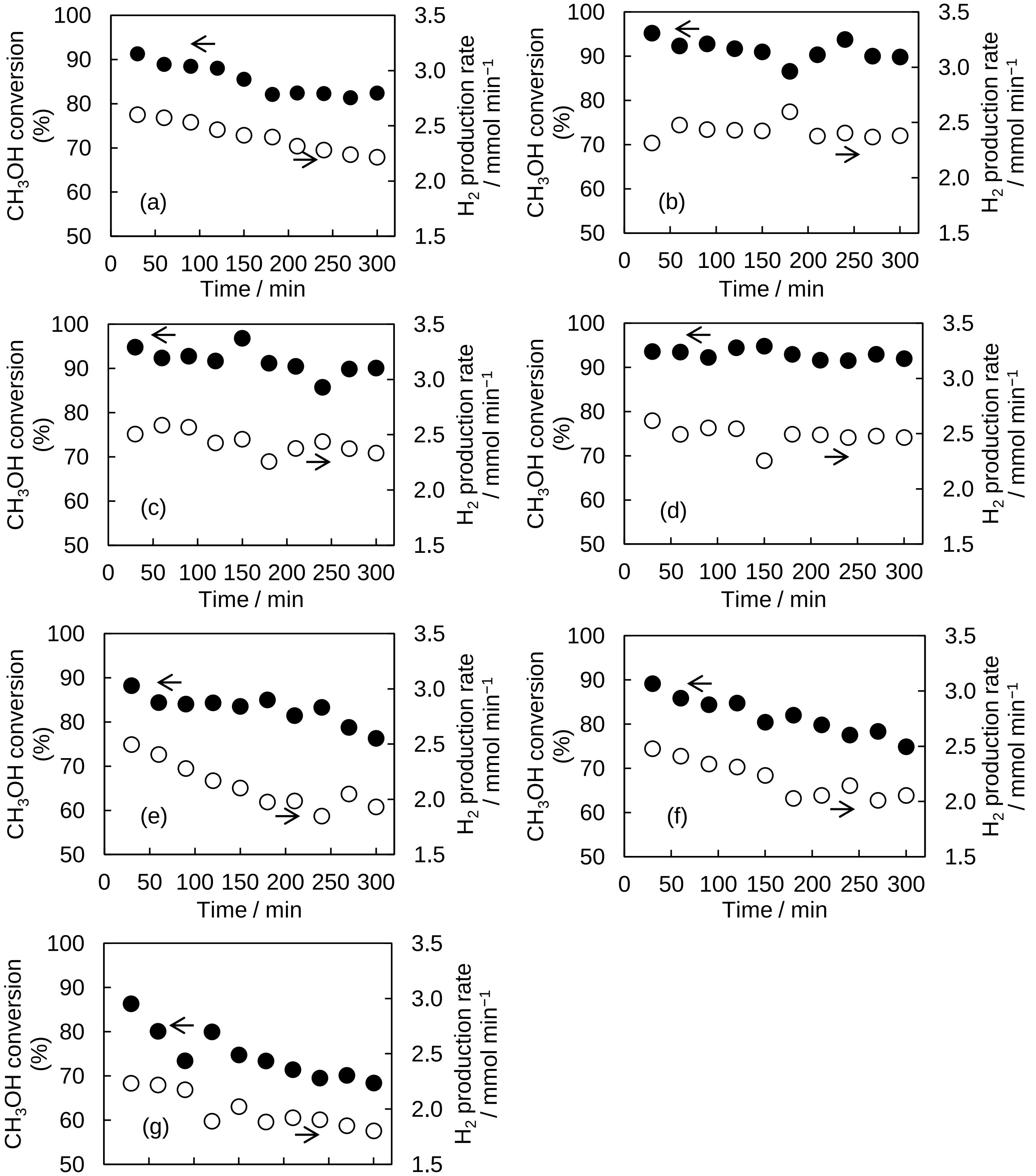

Fig. S2. Time course of methanol conversion (solid) and H_2 production rate (open) in ATR of model bio-methanol (methanol with 1mol% ethanol) over CZA and metal-doped CZA catalysts.

(a) CZA, (b) Rh-CZA, (c) Ru-CZA, (d) Ni-CZA, (e) Pt-CZA, (f) Co-CZA, (g) Pd-CZA.

Reaction conditions: Catalyst 100 mg, T_F 200 °C; $CH_3OH/C_2H_5OH/H_2O/O_2/N_2$=1.23/0.01/1.48/0.41/1.23 mmol min^{-1}.

Table S1. Physical properties of CZA and metal-doped CZA before and after ATR of model bio-methanol (methanol with 1mol% ethanol).

Catalyst	S_{BET} / m^2 g^{-1}	Cu surface are[a)] / m^2 g^{-1}	Cu (111) crystallite diameters[b)] / nm	
	H_2 red.[d)]	H_2 red.[e)]	H_2 red.[d)]	ATR of model bio-methanol[c)]
Rh-CZA	48	24.9	4.5	4.3
Ru-CZA	44	16.2	4.8	6.3
Ni-CZA	48	13.5	3.3	5.0
Pt-CZA	78	13.9	3.1	5.7
Co-CZA	44	13.4	3.6	5.9
Pd-CZA	53	16.8	4.7	8.5
CZA	63	17.6	3.0	7.0

a) Calculated from N_2O pulse. b) Calculated by Scherrer's equation from XRD pattern. c) ATR of model bio-methanol (methanol with 1mol% ethanol) at 200 °C. d) after H_2 reduction (300 °C, 1 h, H_2 10 mL min^{-1}). e) after H_2 reduction (300 °C, 1 h, H_2/He 5/30 mL min^{-1}).

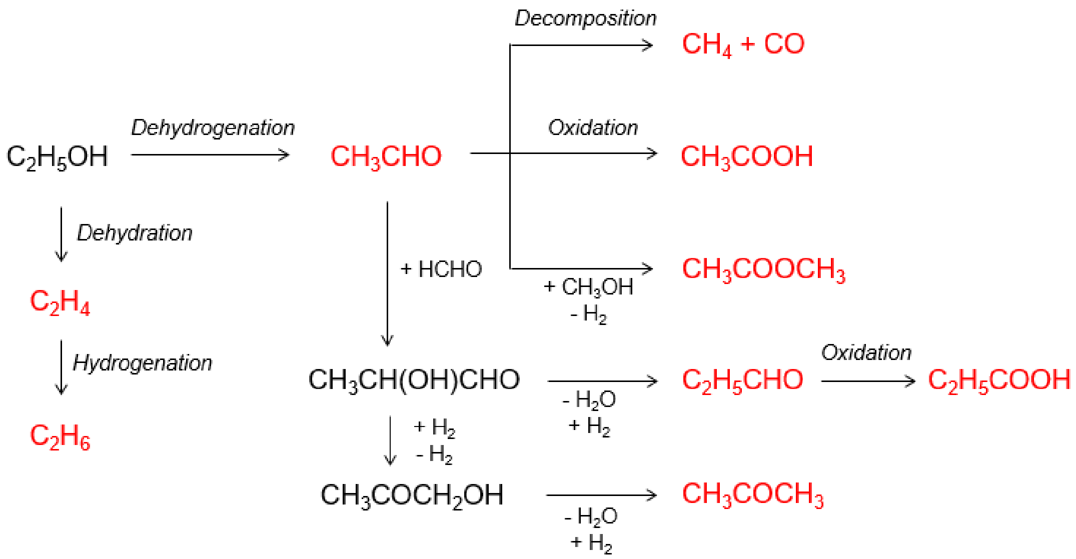

Fig. S3. Proposal side reaction mechanism in reforming of model bio-methanol (methanol with ethanol). The compounds shown in red are produced in ATR of model bio-methanol (methanol with ethanol) as byproducts.

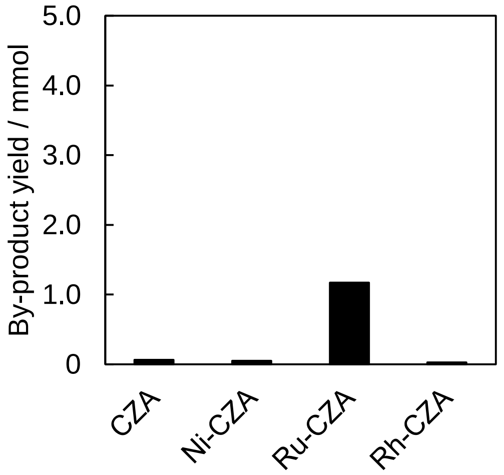

Fig. S4. Methane yield over (a) CZA, (b) Ni-CZA, (c) Ru-CZA and (d) Rh-CZA in ATR of methanol.
Reaction conditions: Catalyst 100 mg, 300 min, T_F 200 °C; $CH_3OH/H_2O/O_2/N_2$=1.23/1.48/0.41/1.23 mmol min^{-1}.

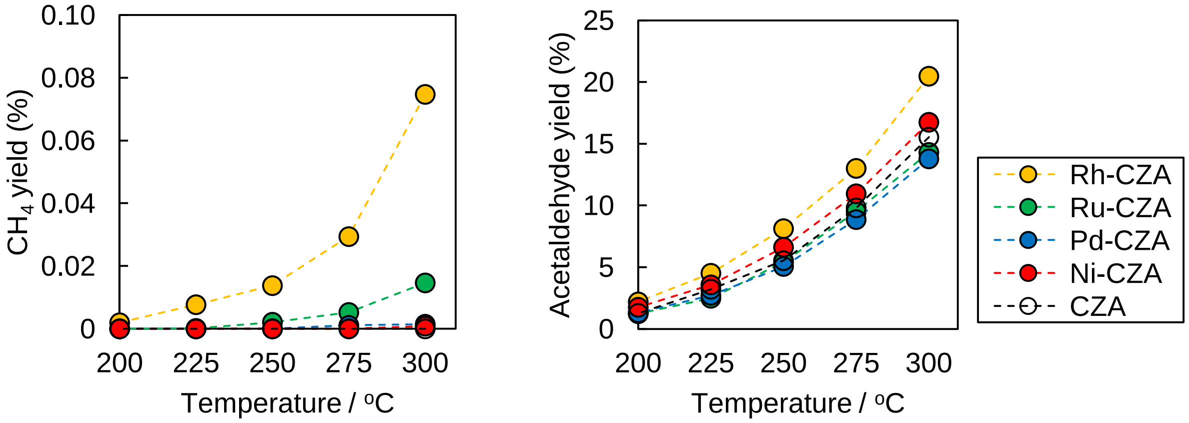

Fig. S5. Ethanol decomposition over CZA and metal-doped CZA.
Reaction conditions: Catalyst 10 mg; C_2H_5OH/N_2=0.82/1.23 mmol min^{-1}.

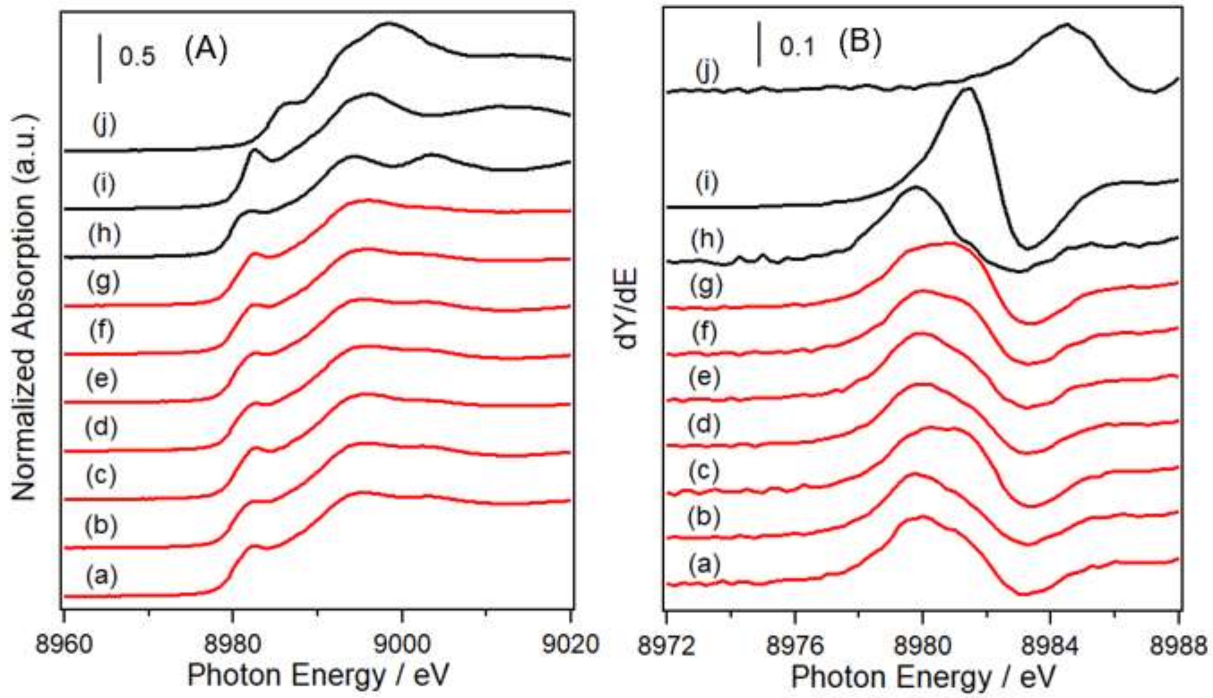

Fig. S6. Cu K-edge XANES Spectra (A) and Their First Derivatives (B) of CZA, metal-doped CZA and References.

(a) CZA, (b) Pd-CZA, (c) Co-CZA, (d) Pt-CZA, (e) Ni-CZA, (f) Ru-CZA, (g) Rh-CZA, (h) Cu foil, (i) Cu_2O, and (j) CuO.

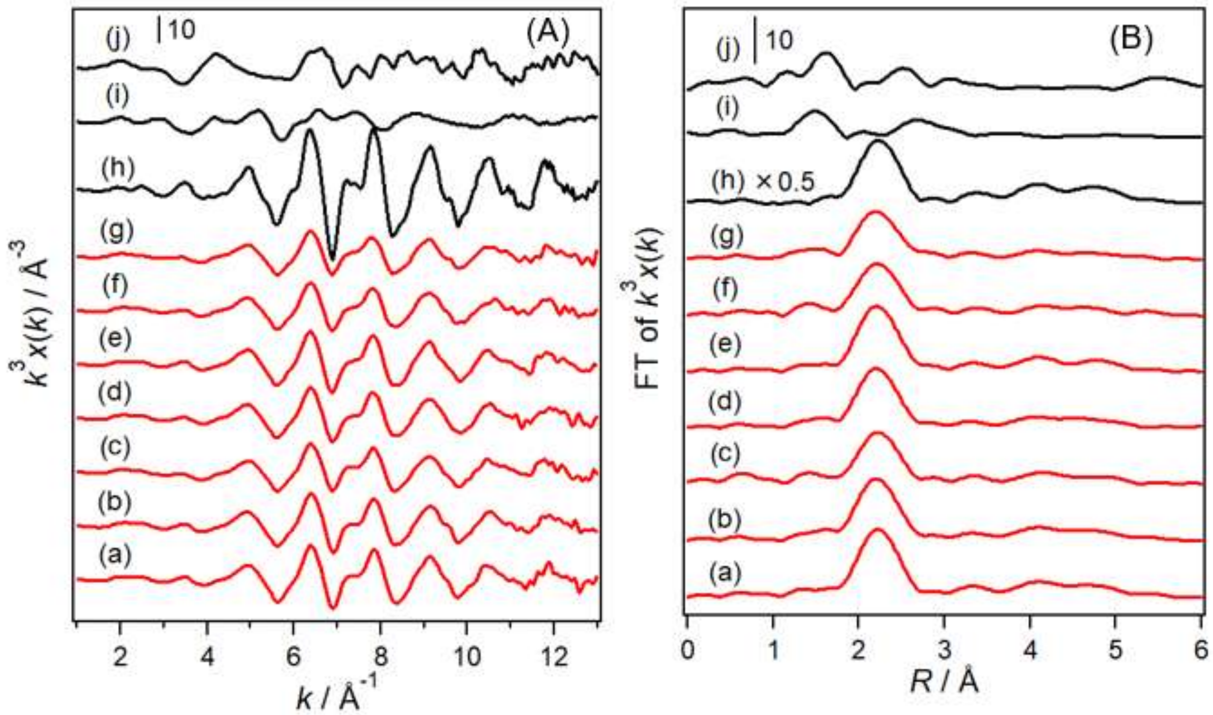

Fig. S7. k^3-weighted Cu K-edge EXAFS Oscillations (A) and Their Fourier Transformations (B) of CZA, metal-doped CZA and References (k=3–13 Å^{-1}).

(a) CZA, (b) Pd-CZA, (c) Co-CZA, (d) Pt-CZA, (e) Ni-CZA, (f) Ru-CZA, (g) Rh-CZA, (h) Cu foil, (i) Cu_2O, and

(j) CuO.

Table S2. Curve-fitting analyses of Cu K-edge EXAFS spectra.

Catalyst[a)]	Scatter	N [b)]	$R^{c)}$ / Å	$\Delta E^{d)}$ / eV	D.W.[e)]
Rh-CZA	Cu	5.1	2.52	1.42	0.102
Ru-CZA	Cu	6.0	2.53	2.42	0.105
Ni-CZA	Cu	6.9	2.52	1.73	0.102
Pt-CZA	Cu	5.9	2.52	1.52	0.100
Co-CZA	Cu	5.5	2.53	3.30	0.102
Pd-CZA	Cu	6.5	2.52	1.98	0.102
CZA	Cu	6.8	2.53	2.92	0.100
Cu foil	Cu	10.6	2.53	2.87	0.093

FEFF8 was used for the calculation of the back-scattering amplitude and phase shift functions [3]. The crystal structures obtained from ICDD (International Centre for Diffraction Data) (Cu (Fm-3m: 00-004-0836)).

a) after H_2 reduction (300 °C, 1 h, H_2 10 mL min^{-1}). b) coordination number. c) distance. d) edge shift. e) Debye-Waller factor.

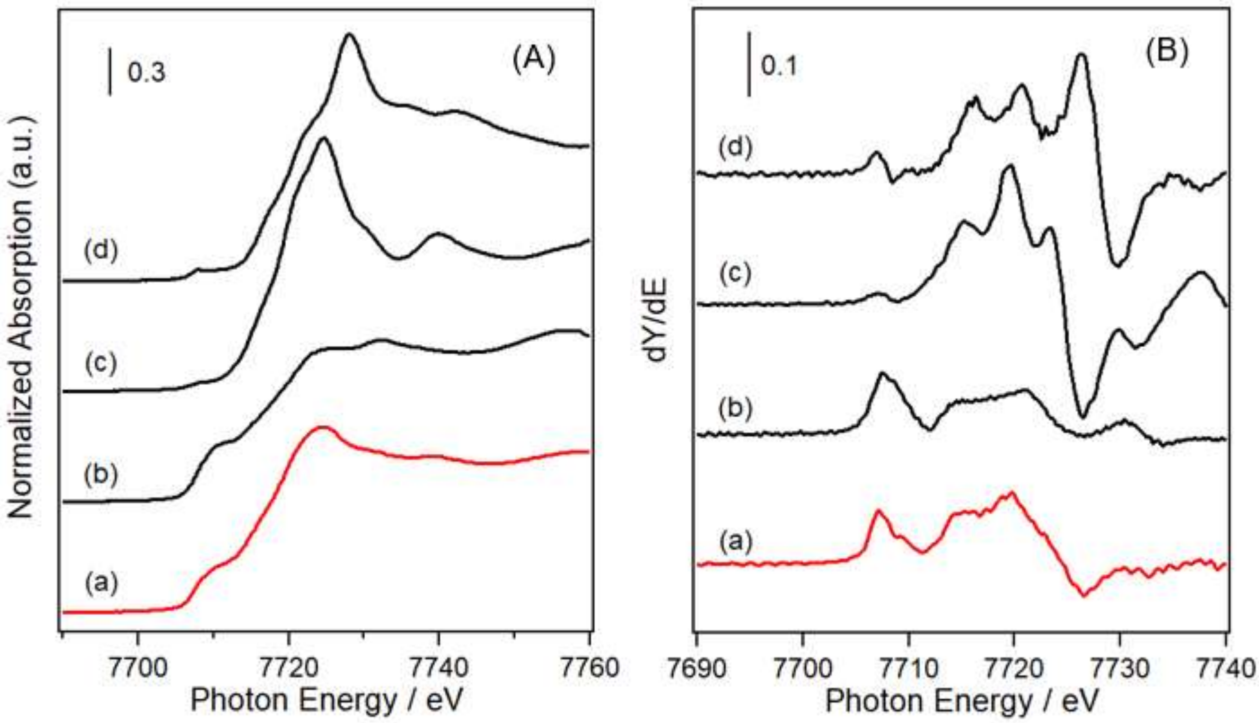

Fig. S8. Co K-edge XANES Spectra (A) and Their First Derivatives (B) of Co-CZA and References.

(a) Co-CZA, (b) Co foil, (c) CoO, (d) Co_3O_4.

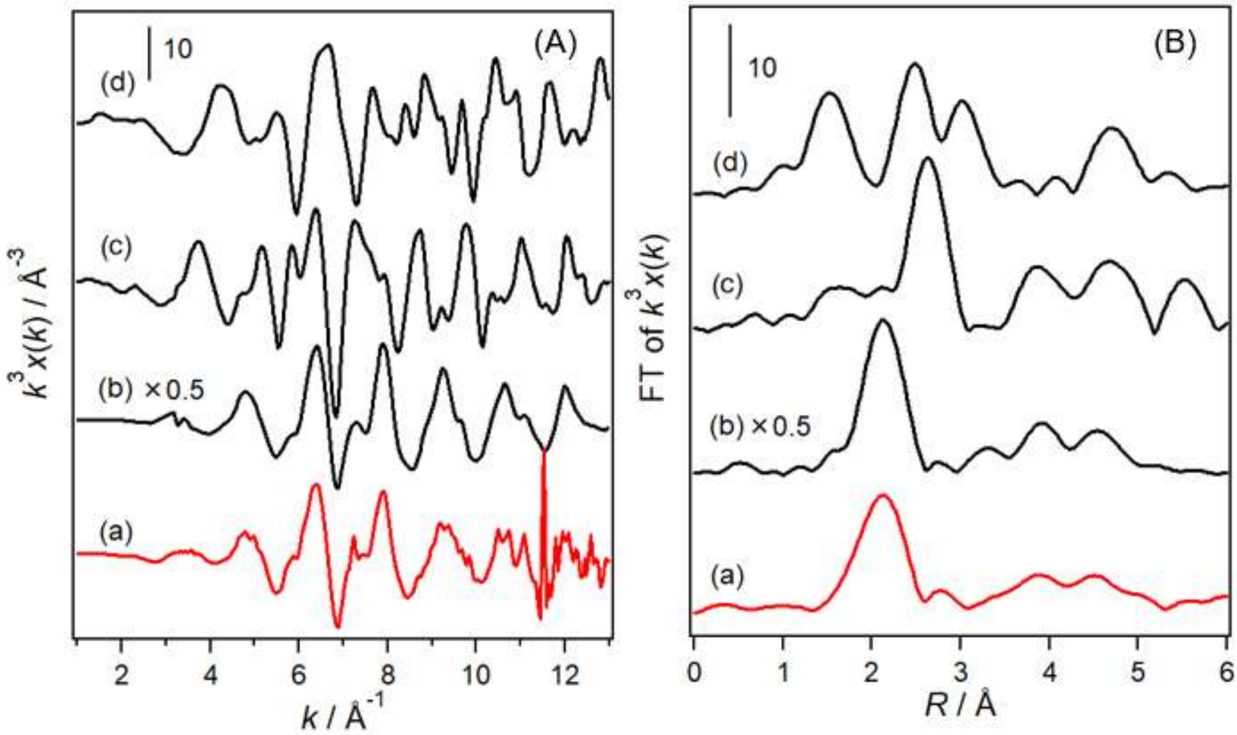

Fig. S9. k^3-weighted Co K-edge EXAFS Oscillations (A) and Their Fourier Transformations (B) of Co-CZA and References (k =3–13 Å^{-1}).

(a) Co-CZA, (b) Co foil, (c) CoO, (d) Co_3O_4.

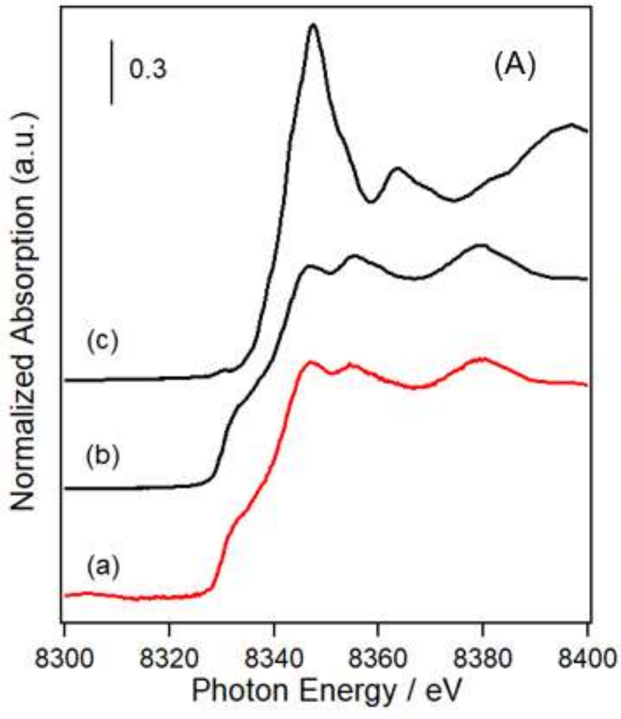

Fig. S10. Ni K-edge XANES Spectra (A) and Their First Derivatives (B) of Ni-CZA and References.
(a) Ni-CZA, (b) Ni foil, (c) NiO.

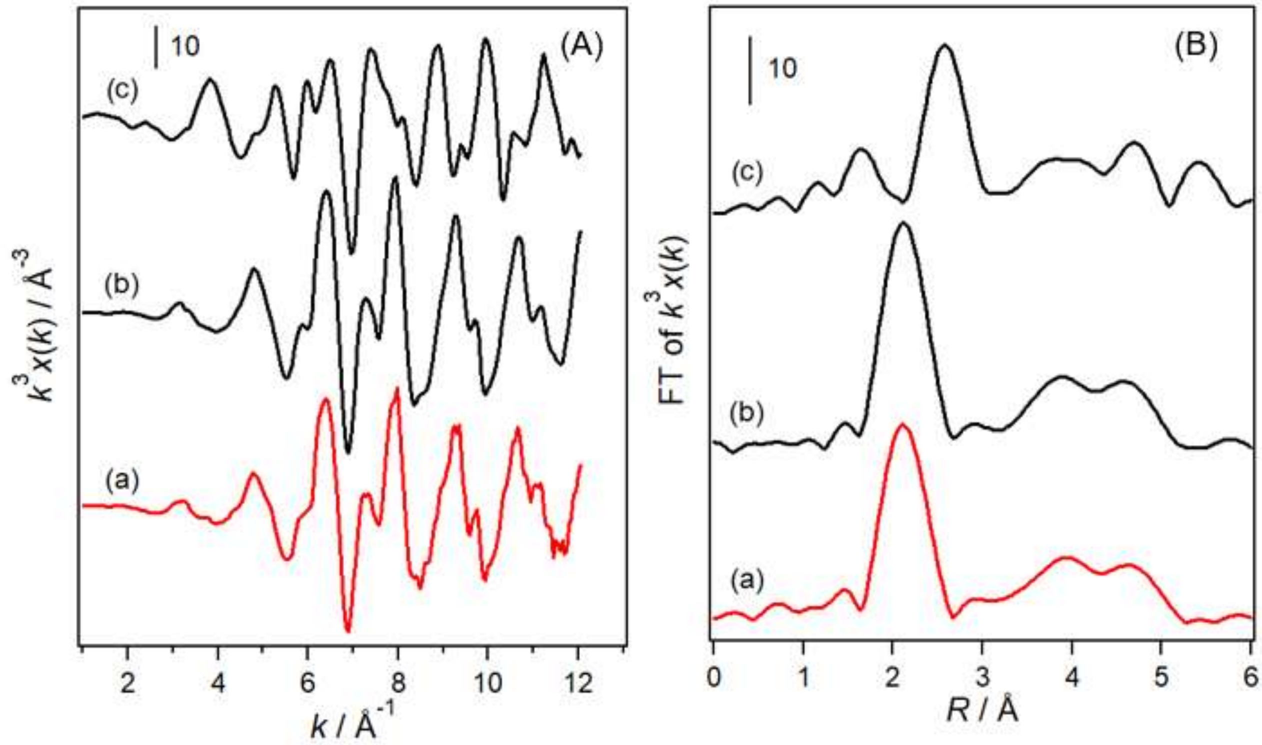

Fig. S11. k^3-weighted Ni K-edge EXAFS Oscillations (A) and Their Fourier Transformations (B) of Ni-CZA and References (k =3–12 Å^{-1}).
(a) Ni-CZA, (b) Ni foil, (c) NiO.

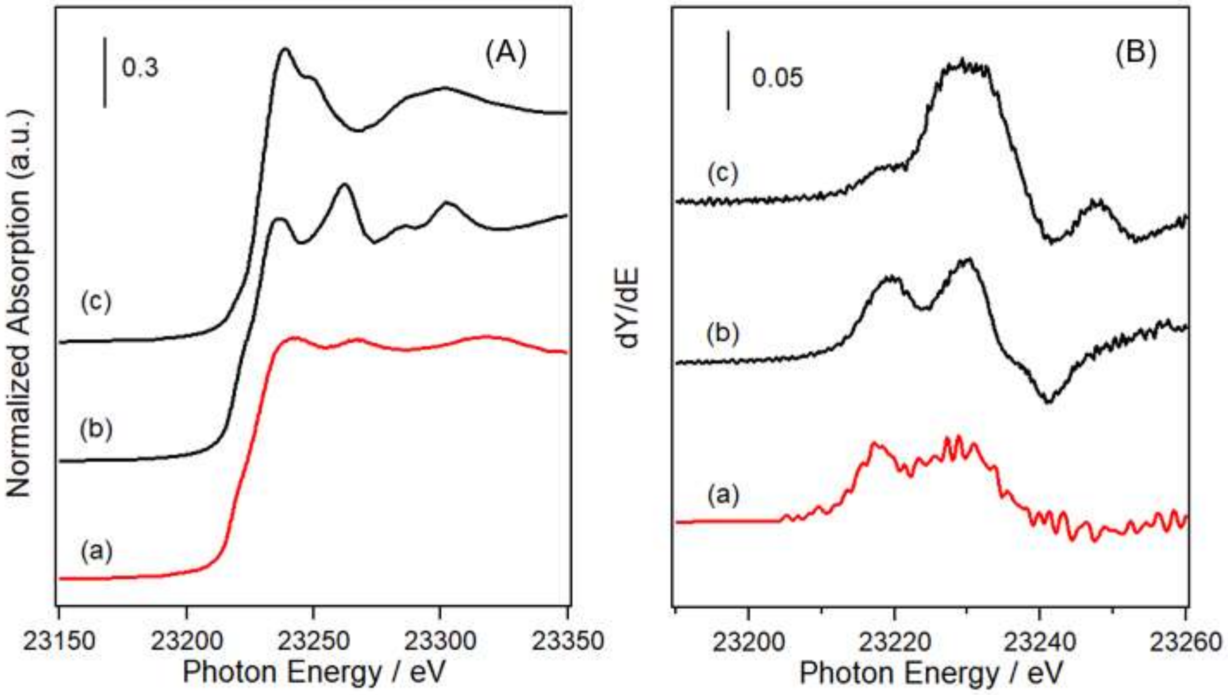

Fig. S12. Rh K-edge XANES Spectra (A) and Their First Derivatives (B) of Rh-CZA and References.

(a) Rh-CZA, (b) Rh foil, (c) Rh_2O_3.

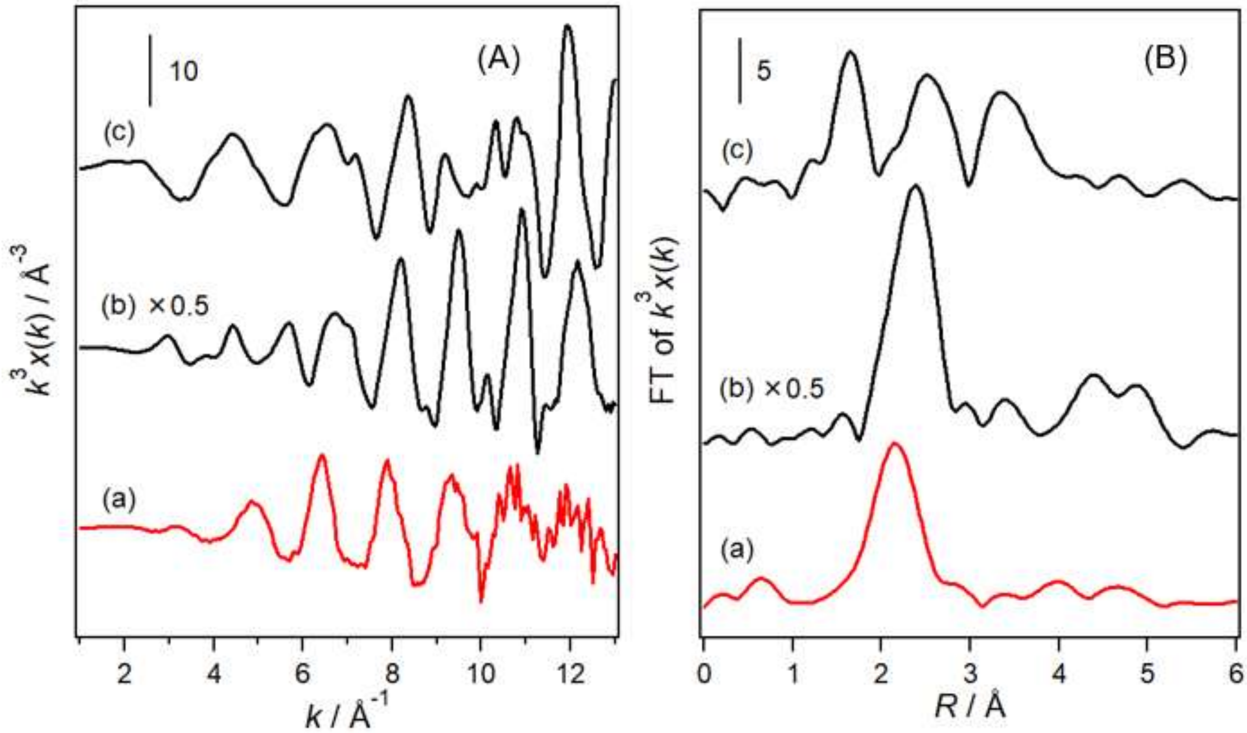

Fig. S13. k^3-weighted Rh K-edge EXAFS Oscillations (A) and Their Fourier Transformations (B) of Rh-CZA and References (k =3–13 Å^{-1}).

(a) Rh-CZA, (b) Rh foil, (c) Rh_2O_3.

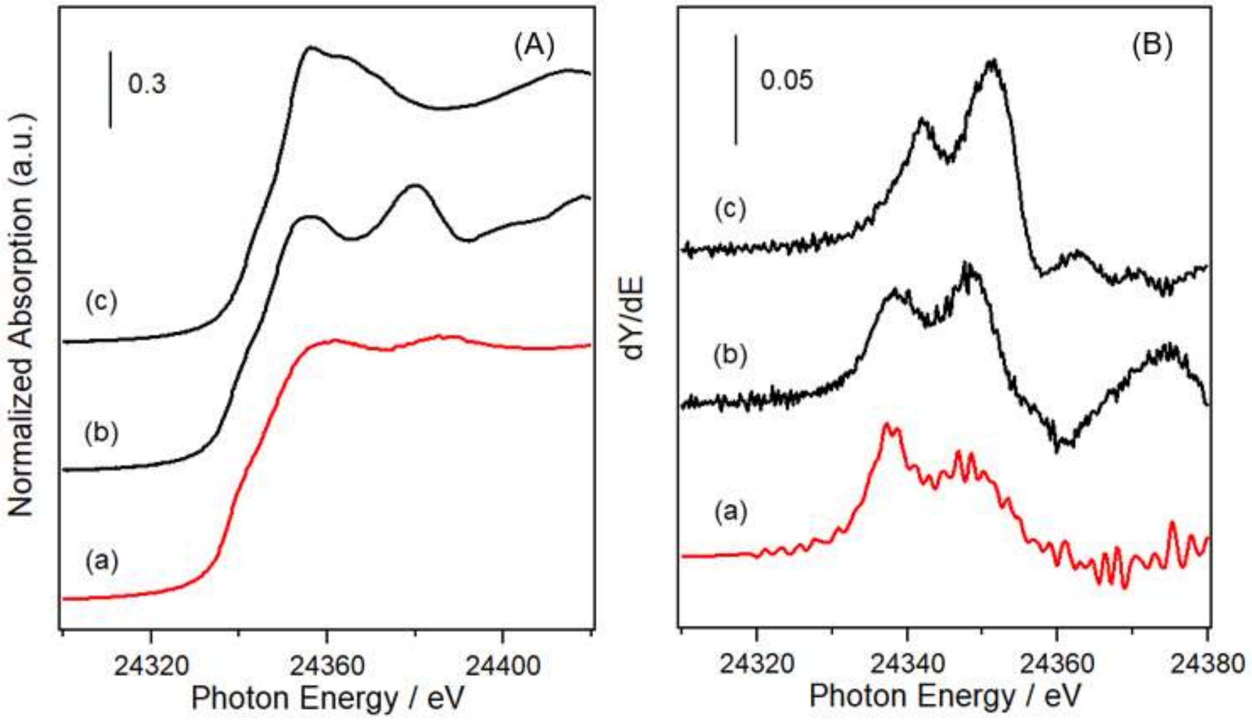

Fig. S14. Pd K-edge XANES Spectra (A) and Their First Derivatives (B) of Pd-CZA and References.

(a) Pd-CZA, (b) Pd foil, (c) PdO.

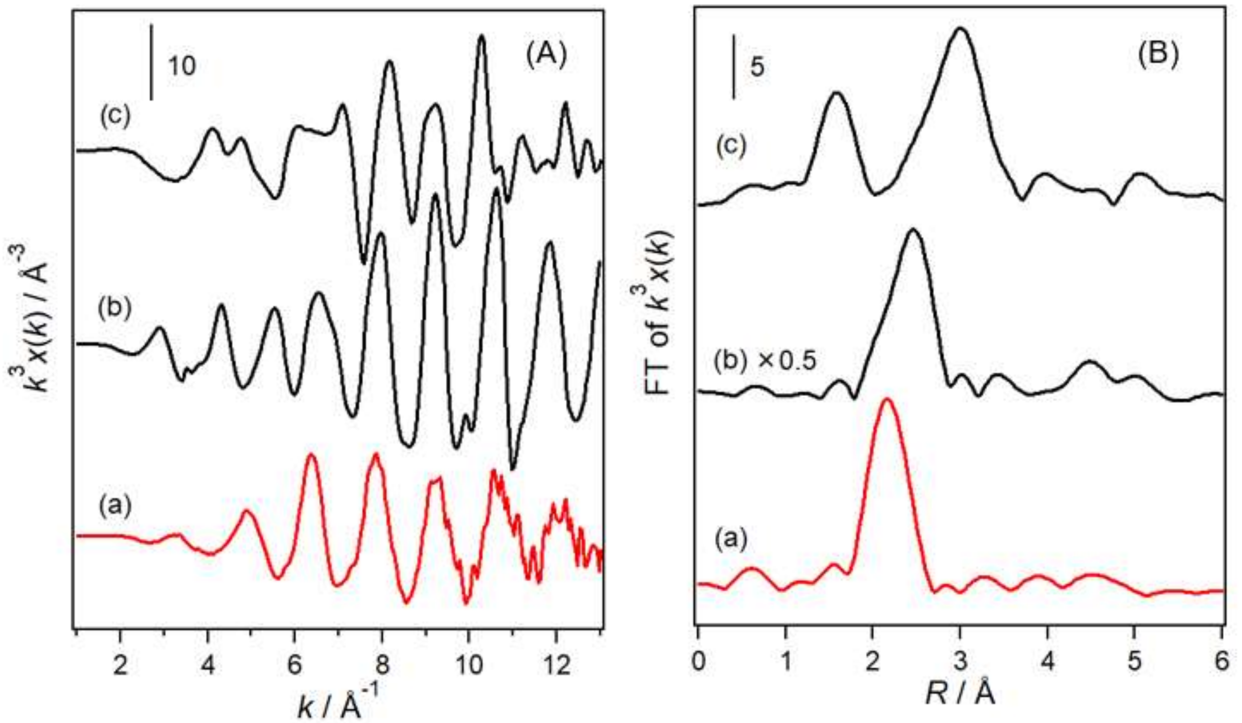

Fig. S15. k^3-weighted Pd K-edge EXAFS Oscillations (A) and Their Fourier Transformations (B) of Pd-CZA and References (k =3–13 Å^{-1}).

(a) Pd-CZA, (b) Pd foil, (c) PdO.

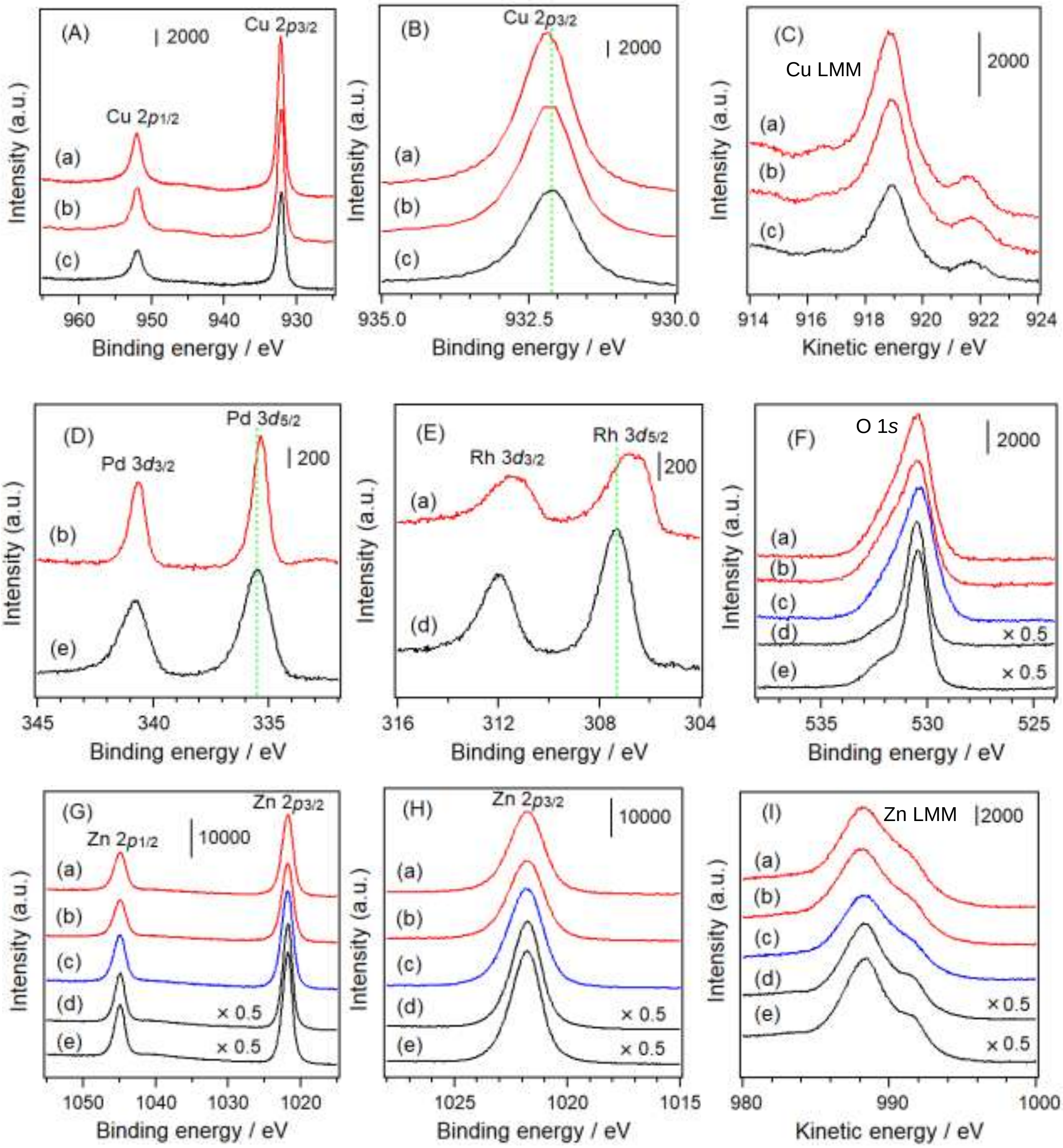

Fig. S16. X-ray photoelectron spectra of (A, B) Cu 2*p*, (C) Cu LMM, (D) Pd 3*d*, (E) Rh 3*d*, (F) O 1*s*, (G, H) Zn 2*p*, and (I) Zn LMM regions of CZA, Pd-CZA, Rh-CZA, and references (Pd/ZnO and Rh/ZnO) after reduction.

(a) Rh-CZA, (b) Pd-CZA, (c) CZA, (d) Rh/ZnO, and (e) Pd/ZnO.

(B) and (H) are the enlarged view of (A) and (G), respectively.

Pretreatment: H_2 reduction (300 or 350 (Rh/ZnO) °C, 0.5 h, H_2/Ar=10/30 mL min^{-1}).

X-ray photoelectron spectroscopy: Al Kα (1486.6 eV), Pass energy 55 eV, Resolution 0.050 eV.

Calibrated by Zn 2$p_{3/2}$ 1021.80 eV [2].

Table S3. Binding energy (Cu 2$p_{3/2}$ and Zn 2$p_{3/2}$), kinetic energy (Cu LMM and Zn LMM), and Auger parameter (α) of CZA, Pd-CZA, and Rh-CZA after reduction.

Sample	Cu 2$p_{3/2}$ / eV	Cu LMM / eV	α_{Cu} / eV	Zn 2$p_{3/2}$ / eV	Zn LMM / eV	α_{Zn} / eV
Rh-CZA	932.19	918.84	1851.03	1021.80	988.20	2010.00
Pd-CZA	932.14	918.91	1851.05	1021.80	988.10	2009.90
CZA	932.11	918.93	1851.04	1021.80	988.21	2010.01

α_{Cu} = Binding energy (Cu 2$p_{3/2}$) + Kinetic energy (Cu LMM) [4,5].

α_{Zn} = Binding energy (Zn 2$p_{3/2}$) + Kinetic energy (Zn LMM) [4,6].

Pretreatment: H_2 reduction (300 °C, 0.5 h, H_2/Ar=10/30 mL min^{-1}).

X-ray photoelectron spectroscopy: Al Kα (1486.6 eV), Pass energy 55 eV, Resolution 0.050 eV.

Calibrated by Zn 2$p_{3/2}$ 1021.80 eV [2].

Table S4. Surface metal composition ratio normalized by Cu concentration of CZA, Pd-CZA, and Rh-CZA after reduction.

Sample	Doped metal (Rh, Pd)	Cu	Zn
Rh-CZA	0.05	1	5.12
Pd-CZA	0.06	1	5.99
CZA	-	1	10.26

Atomic concentration was estimated from XP spectra (Pd 3*d*, Rh 3*d*, Cu 2*p*, Zn 2*p*, O 1*s*, N 1*s*, C 1*s*) (Fig. S16).

Ratio of nominal metal loading: Doped metal/Cu =0.015/1 (Molar ratio) = 1/40.7 (wt%).

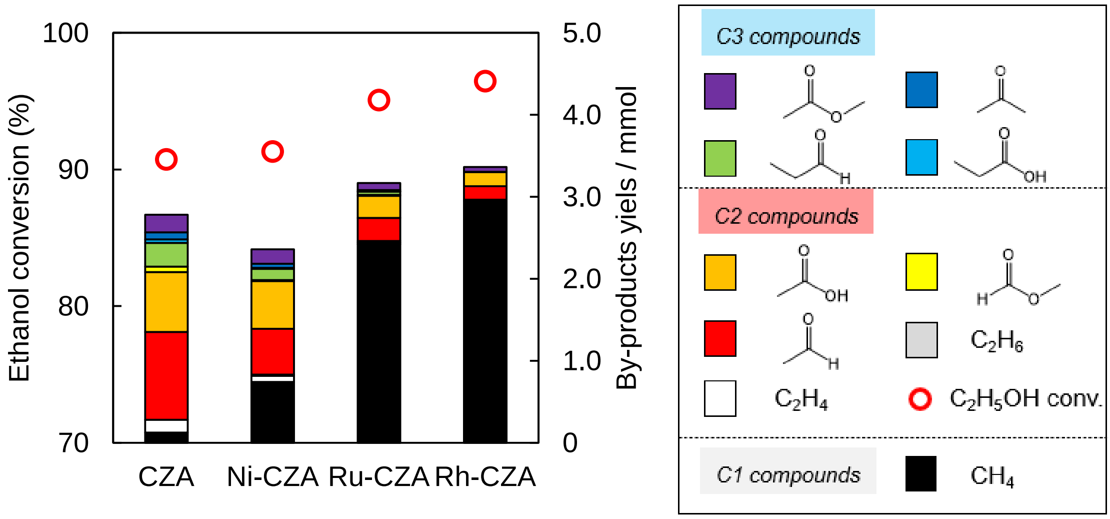

Fig. S17. The yields of by-product and ethanol conversion in ATR of model bio-methanol (methanol with 1mol% ethanol) at 140 °C.

Reaction conditions: Catalyst 100 mg. 300 min; $CH_3OH/C_2H_5OH/H_2O/O_2/N_2$=1.23/0.01/1.48/0.41/1.23 mmol min^{-1}.

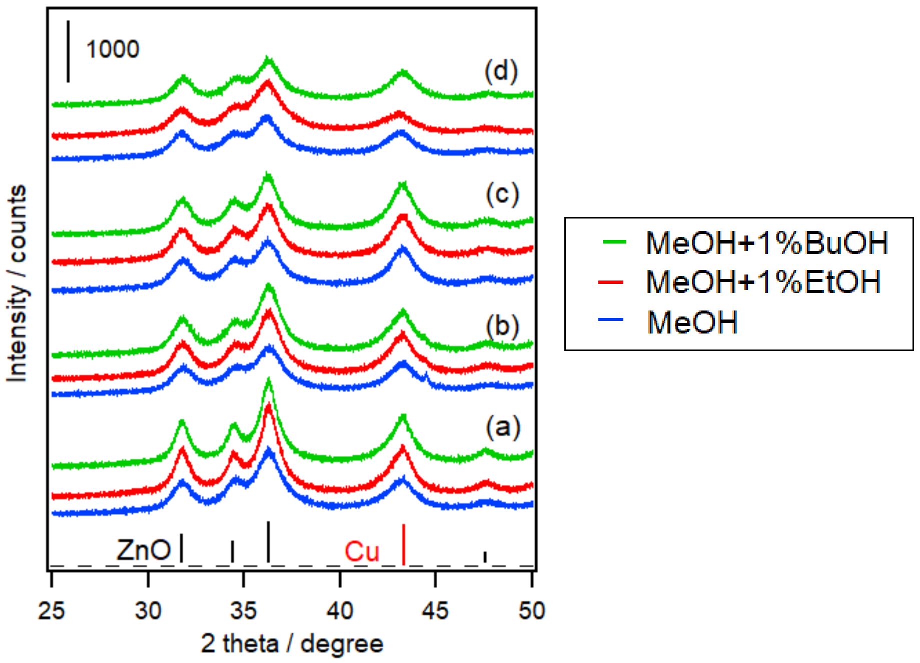

Fig. S18. XRD patterns of spent CZA and metal-doped CZA after ATR of methanol or model bio-methanol (methanol with 1 mol% alcohol).

(a) CZA, (b) Ni-CZA, (c) Ru-CZA, (d) Rh-CZA.

Reaction conditions: Catalyst 100 mg, 300 min, T_F 200 °C; CH_3OH(/Alcohol)/H_2O/O_2/N_2=1.23(/0.01)/1.48/0.41/1.23 mmol min^{-1}.

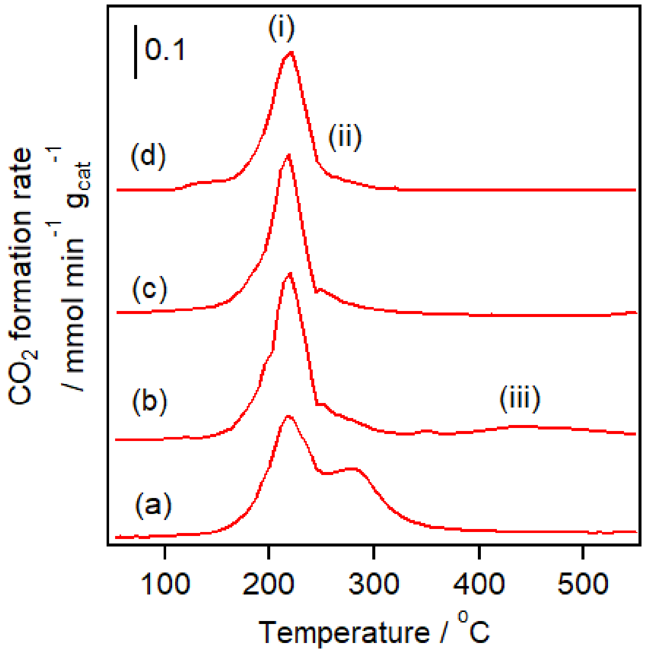

Fig. S19. TPO profiles of spent CZA, Ni-CZA, Ru-CZA and Rh-CZA after ATR of model bio-methanol (methanol with 1mol% butanol).

(a) CZA, (b) Ni-CZA, (c) Ru-CZA, (d) Rh-CZA.

Reaction conditions: Catalyst 100 mg, 300 min, T_F 200 °C; $CH_3OH/C_4H_9OH/H_2O/O_2/N_2$=1.23/0.01/1.48/0.41/1.23 mmol min^{-1}.

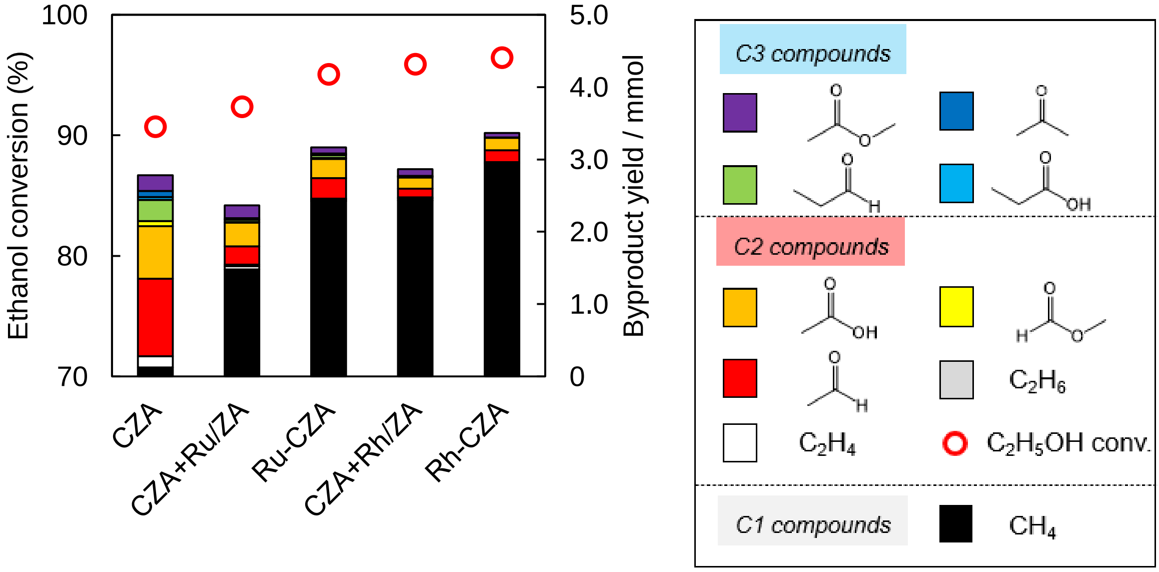

Fig. S20. By-products yield and ethanol conversion over physical mixture in ATR of model bio-methanol (methanol with 1mol% ethanol) at 140 °C.

Reaction conditions: Catalyst: single 100 mg, physical mixture CZA (100 mg)+Ru/ZA or Rh/ZA (100 mg), 300 min; $CH_3OH/C_2H_5OH/H_2O/O_2/N_2$=1.23/0.01/1.48/0.41/1.23 mmol min^{-1}.

Reference

[1] J. W. Evans, M. S. Wainwright, A. J. Bridgewater, D. J. Young, *Appl. Catal.* **1983**, *7*, 75–83.

[2] J. C. Klein, D. M. Hercules, *J. Catal.* **1983**, *82*, 424–441.

[3] A. L. Ankudinov, B. Ravel, J. J. Rehr, S. D. Conradson, *Phys. Rev. B* **1998**, *58*, 7565–7576.

[4] G. Moretti, *J. Electron Spectrosc. Relat. Phenom.* **1998**, *95*, 95–144.

[5] G. Moretti, A. Palma, E. Paparazzo, M. Satta, *Surf. Sci.* **2016**, *646*, 298–305.

[6] L. S. Dake, D. R. Baer, J. M. Zachara, *Surf. Interface Anal.* **1989**, *14*, 71–75.
